# Teneurin-3 Specifies Morphological and Functional Connectivity of Retinal Ganglion Cells in the Vertebrate Visual System

**DOI:** 10.1016/j.celrep.2013.09.045

**Published:** 2013-10-31

**Authors:** Paride Antinucci, Nikolas Nikolaou, Martin P. Meyer, Robert Hindges

**Affiliations:** 1 MRC Centre for Developmental Neurobiology, King’s College London, Guy’s Campus, London SE1 1UL, UK

## Abstract

A striking feature of the CNS is the precise wiring of its neuronal connections. During vertebrate visual system development, different subtypes of retinal ganglion cells (RGCs) form specific connections with their corresponding synaptic partners. However, the underlying molecular mechanisms remain to be fully elucidated. Here, we report that the cell-adhesive transmembrane protein Teneurin-3 (Tenm3) is required by zebrafish RGCs for acquisition of their correct morphological and functional connectivity in vivo. Teneurin-3 is expressed by RGCs and their presynaptic amacrine and postsynaptic tectal cell targets. Knockdown of Teneurin-3 leads to RGC dendrite stratification defects within the inner plexiform layer, as well as mistargeting of dendritic processes into outer portions of the retina. Moreover, a subset of RGC axons exhibits tectal laminar arborization errors. Finally, functional analysis of RGCs targeting the tectum reveals a selective deficit in the development of orientation selectivity after Teneurin-3 knockdown. These results suggest that Teneurin-3 plays an instructive role in the functional wiring of the vertebrate visual system.

## Highlights

• Teneurin-3 is expressed by RGCs, amacrine cells, and tectal neurons
• Teneurin-3 is required by RGC dendrites for correct IPL stratification
• A subset of RGC axons show laminar arborization defects upon Teneurin-3 knockdown
• Orientation-, but not direction-selective, RGCs are impaired in Teneurin-3 morphants

## Graphical Abstract

**Figure:**
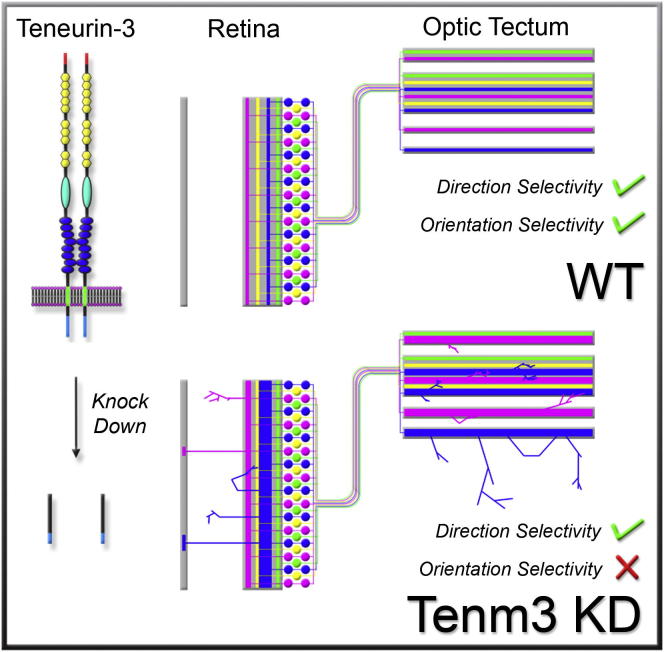

## Teaser

In the vertebrate visual system, different subtypes of retinal ganglion cells (RGCs) form specific connections with their corresponding synaptic partners. In this study, Hindges and colleagues show that knockdown of the cell-adhesive transmembrane protein Teneurin-3 leads to structural defects in subsets of zebrafish RGCs in both their pre- and postsynaptic target regions. Moreover, Teneurin-3 is required for functional development of orientation-selective RGCs. These findings suggest that Teneurin-3 plays an instructive role in wiring cell-subtype-specific visual circuits.

## Introduction

In the vertebrate retina, retinal ganglion cells (RGCs) develop stereotypic dendritic arborization patterns and make specific synaptic connections with amacrine and bipolar cells in the inner plexiform layer (IPL) (Masland, 2012). The formation of such precise connections is critical for the processing of visual information and the generation of feature selectivity in RGCs (Gollisch and Meister, 2010; Wässle, 2004). A key structural characteristic of visual circuits is the organization of connections into precise laminae (Roska and Werblin, 2001; Sanes and Zipursky, 2010). Recent studies have shown that the assembly of neuropil strata in the IPL is regulated by both adhesive (Yamagata and Sanes, 2008) and repulsive transmembrane proteins (Matsuoka et al., 2011). Similarly, such attractive and repulsive cues are also crucial in establishing specific connectivity between RGC axons and their targets in the brain (Osterhout et al., 2011; Xiao et al., 2011). Our understanding of the molecular mechanisms that specify connections within the retina and between the retina and retinorecipient nuclei in the brain, however, is still far from complete.

Teneurins (Ten-m/Odz) are a phylogenetically conserved family of type II transmembrane proteins (Tucker et al., 2012; Tucker and Chiquet-Ehrismann, 2006). Their large extracellular domain contains eight epidermal growth factor (EGF)-like repeats, multiple tyrosine and aspartate (YD) repeats and five NHL (NCL-1, HT2A, and Lin-41) repeats, which mediate homophilic recognition and adhesion (Beckmann et al., 2013). In vertebrates, these proteins are encoded by four genes, *teneurin 1–4* (also called *odz1–4*), expressed in distinct and often interconnected regions of the nervous system (Tucker and Chiquet-Ehrismann, 2006). In *Drosophila*, the role of teneurins in synaptic partner matching and target choice has been elegantly shown in the olfactory system (Hong et al., 2012) and at the neuromuscular junction (Mosca et al., 2012). In mice, teneurins regulate the generation of binocular visual circuits by controlling the development of ipsilaterally projecting RGCs (Dharmaratne et al., 2012; Leamey et al., 2007; Young et al., 2013). However, a role for teneurins in mediating synapse-specific functional wiring in the vertebrate visual system has yet to be demonstrated.

Here, we investigate the role of *teneurin-3* (hereafter referred to as *tenm3*) in shaping the morphological and functional connectivity of RGCs in vivo using zebrafish. We report that *tenm3* is expressed in RGCs, amacrine cells, and the main retinorecipient target in the brain, the optic tectum. We show that *tenm3* knockdown induces stratification and targeting errors of both dendrites and axons in a subset of RGCs. In support of this, we provide evidence showing that orientation-selective, but not direction-selective, responses are impaired in *tenm3* morphants, suggesting that *tenm3* is involved in wiring subsets of functionally defined visual circuits.

## Results

Our study focused on time points between 2 days postfertilization (dpf) and 5 dpf, a period during which RGCs undergo a rapid phase of morphological and functional development (Lowe et al., 2013; Meyer and Smith, 2006; Mumm et al., 2006).

### Teneurin-3 Is Expressed in Interconnected Regions of the Developing Visual System

**Figure 1:**
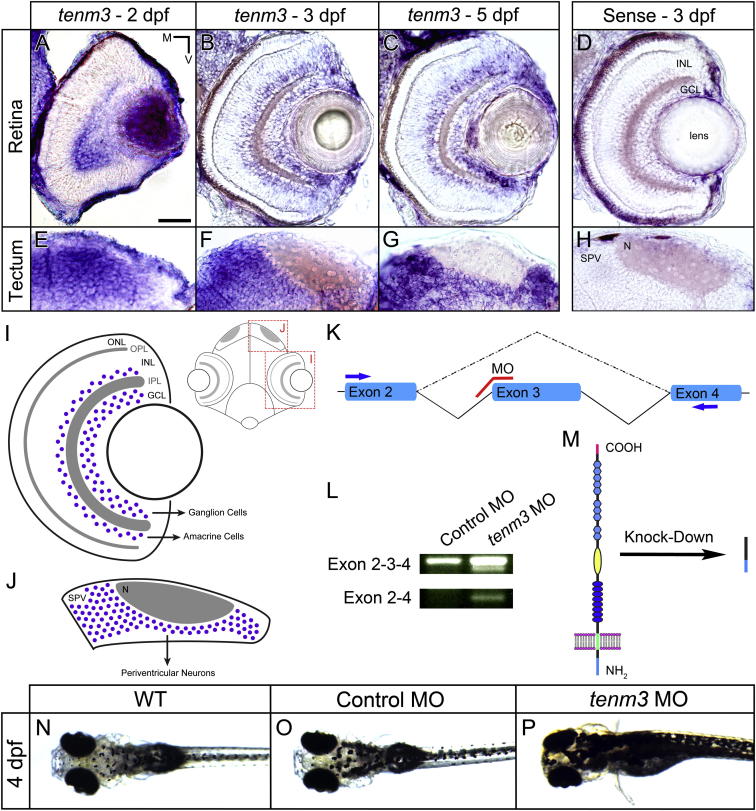
*Teneurin-3* Is Expressed in Interconnected Regions of the Zebrafish Visual System (A–C) Retinal cryosections of whole-mount in situ hybridizations showing *tenm3* mRNA expression at 2, 3, and 5 dpf. (D and H) Control in situ hybridizations using sense *tenm3* riboprobe. (E–G) Tectal cryosections of whole-mount in situ hybridizations showing *tenm3* mRNA expression at 2, 3, and 5 dpf. All images are in transverse plane. Scale bar, 40 μm. N, neuropil; M, medial; V, ventral. (I) Schematic showing the expression pattern of *tenm3* in the retina. *Tenm3*-positive cells are represented as blue circles. Neuropil layers are indicated in gray. Anatomical reference is reported on the right. IPL, inner plexiform layer; ONL, outer nuclear layer; OPL, outer plexiform layer. (J) Schematic showing the expression pattern of *tenm3* in the optic tectum. (K) Schematic detailing the targeting site of splice-blocking *tenm3* morpholino (MO), which is shown in red. Exons are represented in cyan. Solid lines indicate introns. The dashed line indicates exon 3 deletion caused by *tenm3* MO injections. Primers used for RT-PCR (L) are reported as blue arrows. (L) RT-PCR analysis of *tenm3* mRNA structure in control MO- and *tenm3* MO-injected embryos. Two shorter splice variants are distinguished in *tenm3* morphants. cDNA sequence comparison revealed that the shortest splice variant lacks exon 3. (M) Schematic detailing the effect of exon 3 deletion caused by the splice-blocking *tenm3* MO, resulting in the deletion of Tenm3 transmembrane and extracellular domains. The full-length protein is represented on the left. The N terminus is located intracellularly, whereas the C terminus is in the extracellular space. (N–P) At 4 dpf, *tenm3* morphant larvae fail to visually adapt their skin pigmentation to the level of background illumination. See also Figure S1.

To reveal the expression pattern of *tenm3* in the developing zebrafish visual system, we carried out in situ hybridization analyses using a specific digoxigenin-labeled antisense riboprobe against *tenm3*. In the retina, *tenm3* is expressed in the ganglion cell layer (GCL) and the inner third of the inner nuclear layer (INL), where amacrine cells are located (Figures 1A–1C). Since in zebrafish only a very small number of displaced amacrine cells reside in the GCL (Connaughton et al., 1999), the majority of signal detected in this layer can be attributed to RGCs. At 2 dpf, *tenm3* is expressed more strongly in the ventral part of the retina (Figure 1A). At 3 and 5 dpf, *tenm3* acquires a sparse expression pattern, suggesting that at these stages of development only a subset of cells are *tenm3*-positive (Figures 1B and 1C). *Tenm3* is also expressed in the main target of RGC axons, the optic tectum (Figures 1E–1G). At 2 dpf, *tenm3* is highly expressed in the medial portion of the stratum periventriculare (SPV), where cell bodies of most tectal cells are located (Figure 1E). Between 3 and 5 dpf, this medial-to-lateral gradient gradually decreases (Figures 1F and 1G) and, at 5 dpf, *tenm3* shows a salt-and-pepper expression pattern (Figure 1G). In summary, *tenm3* is expressed by RGCs, amacrine cells, and tectal neurons (Figures 1I and 1J), consistent with a possible role of *tenm3* in instructing connectivity along the visual pathway.

### Teneurin-3 Regulates RGC Dendritic Stratification in the IPL

To investigate the function of *tenm3* within the developing visual system, we used antisense morpholino oligonucleotides (MOs) to knock down *tenm3* expression levels. We designed a splice-blocking MO (Draper et al., 2001) targeting the boundary between intron 2 and exon 3 (hereafter referred to as *tenm3* MO; Figure 1K). Injection of *tenm3* MO into one-cell-stage zebrafish embryos produces the deletion of exon 3 (Figure 1L), which encodes part of the intracellular domain. This leads to a frameshift in exon 4 (transmembrane domain) and a subsequent early stop codon in exon 5, resulting in deletion of the transmembrane and extracellular domains (Figure 1M). To confirm results obtained with this *tenm3* MO, a second splice-blocking MO targeting a nonoverlapping region of *tenm3* mRNA (i.e., the boundary between exon 4 and intron 4) was also used (*tenm3* MO 2; see Figure S1). *Tenm3* morphants are viable and do not show any obvious morphological defect. However, 4 dpf *tenm3* MO-injected larvae fail to show a normal visually mediated background adaptation (VBA) and therefore appear darker compared to wild-type (WT) and control MO-injected larvae (Figures 1N–1P). Since the VBA is a neuroendocrine response dependent on the function of RGCs (Kay et al., 2001), we deduced that the knockdown of *tenm3* somehow impairs the normal development of the visual system.

**Figure 2:**
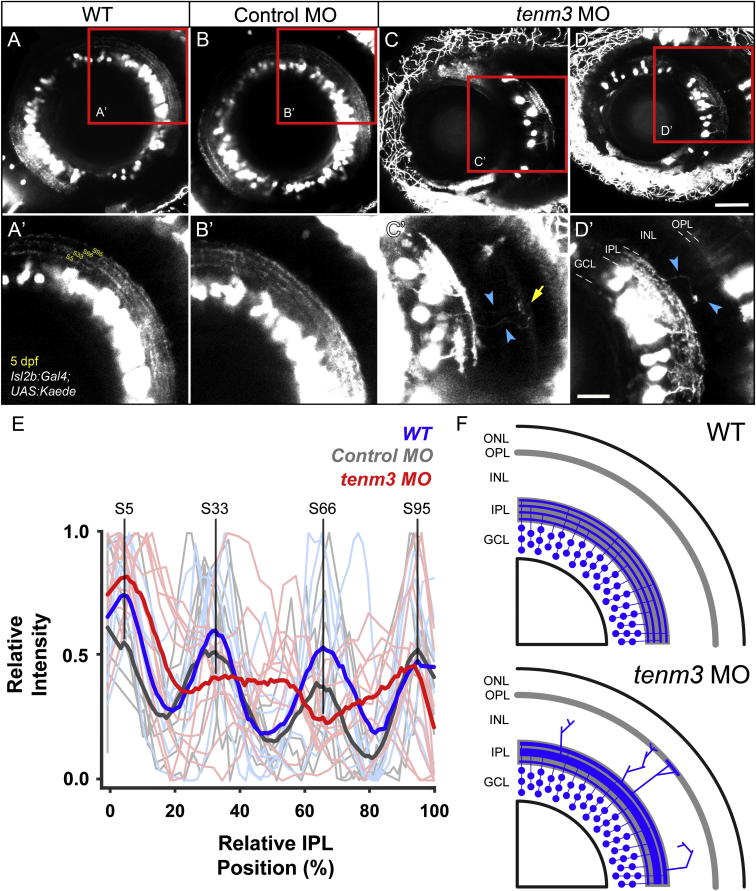
*Teneurin-3* Is Required for Correct Stratification of RGC Dendrites (A–D) Kaede-expressing RGCs in the retina of 5 dpf WT, control MO-injected, and *tenm3* MO-injected larvae. (A′–D′) Insets in (A)–(D) showing the dendritic stratification pattern of Kaede-positive RGCs. All images represent maximum intensity projections of ∼20 μm confocal z stacks. Scale bars, 40 μm (A–D) and 20 μm in (A′–D′). GCL, ganglion cell layer; INL, inner nuclear layer; IPL, inner plexiform layer; OPL, outer plexiform layer. (E) Fluorescence profiles of IPL stratification in 5 dpf WT (blue), control MO-injected (gray), and *tenm3* MO-injected (red) larvae. Thin traces represent intensity profiles of IPLs of single larvae. Thick traces indicate average profiles (WT, n = 7 larvae; control MO, n = 7; *tenm3* MO, n = 10). Zero percent corresponds to the boundary between GCL and IPL, whereas 100% corresponds to the boundary between IPL and INL. (F) Schematic summarizing the defects observed in *tenm3* morphant retinae. RGCs are indicated in blue. Neuropil layers are in gray. ONL, outer nuclear layer. See also Figure S4.

In order to examine IPL organization in vivo, we used the Tg(*Isl2b:Gal4;UAS:Kaede*) transgenic zebrafish line (see Experimental Procedures), where the fluorescent protein Kaede is expressed in the majority of RGCs. At 5 dpf, when RGC dendrites exhibit clear stratification, four Kaede-positive strata are visible in the IPL of WT and control MO-injected larvae (Figures 2A and 2B). Fluorescence intensity measurements across the IPL of multiple larvae show that these strata are positioned at 5%, 33%, 66%, and 95% depth of the IPL (with 0% corresponding to GCL/IPL border and 100% to IPL/INL border), and thus were named S5, S33, S66, and S95, respectively (Figure 2E; WT n = 7 larvae, control MO n = 7). The presence of four dendritic strata in the IPL of 5 dpf zebrafish larvae is consistent with previous work using the Tg(*Brn3c:MGFP*) transgenic line, where approximately 50% of RGCs are labeled (Mumm et al., 2006). In 5 dpf *tenm3* morphants, by contrast, strata within the IPL are poorly defined (Figures 2C and 2D). The average fluorescence intensity profile reveals that only three Kaede-positive strata are present in the IPL of *tenm3* morphants (Figure 2E; n = 10 larvae). Specifically, only one irregularly laminated stratum is visible in the medial portion of the IPL, instead of the two middle strata (S33 and S66) found in WT and control MO retinae. Furthermore, the outermost stratum (S95) is not tightly stratified and appears thicker compared to control groups. No significant difference in IPL width was observed among the three groups (WT 15.2 ± 0.2 μm; control MO 15.0 ± 0.1 μm; *tenm3* MO 15.1 ± 0.2 μm; F_2,21_ = 0.08, p = 0.92, n = 24 larvae). In addition to these stratification abnormalities in the IPL, we detected ectopic RGC processes in the INL of *tenm3* morphants (Figures 2C′ and 2D′, cyan arrowheads; n = 19 out of 20 larvae), a phenomenon never observed in WT and control MO larvae, where all RGC dendrites are confined within the IPL (Figures 2A′ and 2B′; n = 10 larvae per group). Strikingly, in some cases, these processes reach the outer plexiform layer (OPL; Figure 2C′, yellow arrow). Ectopic RGC processes extending into the INL were also seen in *tenm3* morphant retinae at 3 dpf, when RGCs start to develop stratified dendritic arbors within the IPL (data not shown).

**Figure 3:**
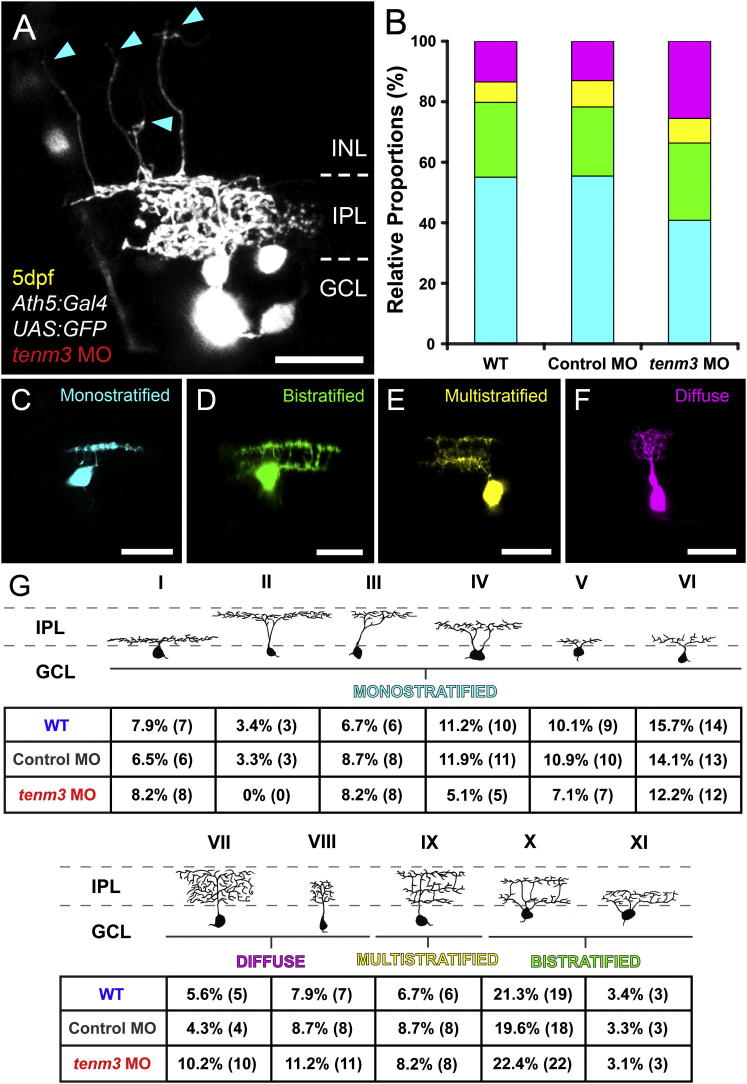
Higher Proportion of RGCs with Diffuse Dendritic Arbors in *teneurin-3* Morphants (A) Lateral view of mosaically labeled RGCs in the retina of a 5 dpf *tenm3* MO-injected larva. Scale bar, 20 μm. GCL, ganglion cell layer; INL, inner nuclear layer; IPL, inner plexiform layer. (B) Bar graph showing the proportions of 5 dpf RGCs possessing monostratified (cyan, C), bistratified (green, D), multistratified (yellow, E), and diffuse (magenta, F) dendritic arbors relative to the total number mosaically labeled RGCs within each animal group (WT n = 89 cells in 34 larvae; control MO n = 92 cells in 39 larvae; *tenm3* MO n = 98 cells in 49 larvae). (C–F) Representative RGCs with monostratified (C), bistratified (D), multistratified (E), and diffuse (F) dendritic arbors. All images represent maximum intensity projections of ∼30 μm confocal z stacks that have been pseudocolored and rotated to best show dendritic arborizations. Scale bars, 20 μm. (G) Summary table showing the morphological classification and frequency of the 11 RGC types within each group (number of cells found per each type are reported in brackets). In *tenm3* morphants, four diffuse RGCs (4.1% of cells) showed dendritic arborization patterns that could not be classified in any of the 11 types and, hence, were not included in the table.

To resolve the changes in RGC dendritic morphology in greater detail, we mosaically labeled individual RGCs by coinjecting *Ath5:Gal4*, *UAS:GFP* and *UAS:tdTomato* DNA constructs into one-cell-stage embryos. The combinatorial expression of different fluorescent reporters in RGCs enabled us to distinguish between occasionally overlapping dendritic arbors of different cells. Using this approach, we were able to determine that the neurites mistargeting into outer layers of the retina observed in *tenm3* morphants originate from RGC dendrites (Figure 3A, cyan arrowheads) and that this phenotype is restricted to a subset of cells (n = 5 cells out of 98 in 49 larvae). Moreover, mosaic labeling allowed us to visualize the precise IPL dendritic stratification patterns of single RGCs (Figures 3C–3F). Interestingly, 5 dpf *tenm3* morphants show a significantly higher proportion of RGCs possessing diffuse dendritic arbors (*tenm3* MO 25 diffuse versus 73 stratified cells in 49 larvae; WT 12 diffuse versus 77 stratified cells in 34 larvae; control MO 12 diffuse versus 80 stratified cells in 39 larvae; χ^2^ = 6.596, df = 2, p = 0.037). Looking at the relative proportions between monostratified, bistratified, multistratified, and diffuse RGCs, it appears that the increase in number of RGCs with diffuse dendritic arbors is exclusively at the expense of monostratified RGCs (Figure 3B; WT 55.1% monostratified, 24.7% bistratified, 6.7% multistratified, 13.5% diffuse; control MO 55.5% monostratified, 22.8% bistratified, 8.7% multistratified, 13% diffuse; *tenm3* MO 40.8% monostratified, 25.5% bistratified, 8.2% multistratified, 25.5% diffuse). Further identification and classification of the 11 RGC types previously reported in the adult zebrafish retina (Mangrum et al., 2002) revealed that the monostratified RGC types are not indiscriminately affected by *tenm3* knockdown. In fact, some RGC monostratified types decrease in frequency in *tenm3* morphants whereas others show frequencies comparable to those found in control animals (Figure 3G). Overall, these data show that *tenm3* knockdown causes structural irregularities in the developing retina (Figure 2F) and that changes in RGC dendritic stratification appear to be limited to specific RGC types.

### Laminar Targeting Errors in a Subset of RGC Axons Upon Teneurin-3 Knockdown

**Figure 4:**
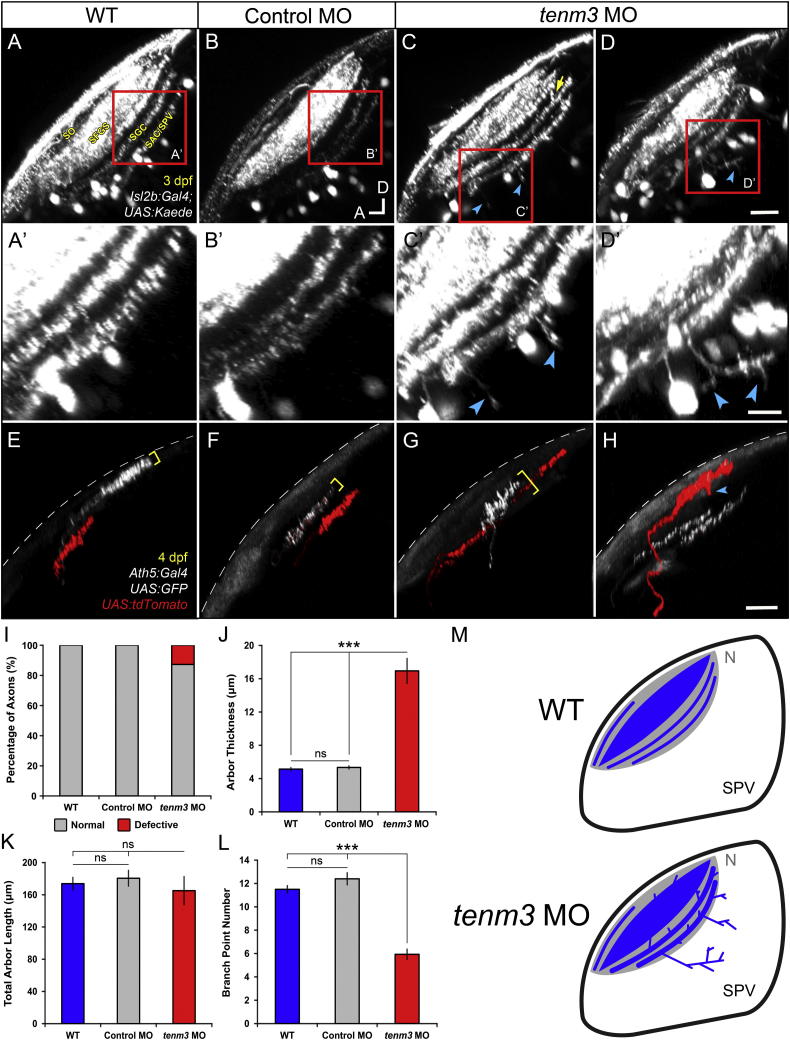
Axon Laminar Targeting Errors in a Subset of RGCs in *teneurin-3* Morphants (A–D) The four main retinorecipient laminae of the tectum are visible in the Tg(*Isl2b:Gal4;UAS:Kaede*) zebrafish line at 3 dpf. SO, stratum opticum; SFGS, stratum fibrosum et griseum superficiale; SGC, stratum griseum centrale; SAC, stratum album centrale; SPV, stratum periventriculare. (A′–D′) Insets in (A)–(D) showing RGC axon lamination in deep laminae of the tectal neuropil. (E–H) Lateral view of mosaically labeled RGC axons at 4 dpf. Dashed lines indicate the skin overlaying the tectum. All images represent maximum intensity projections of ∼50 μm confocal z stacks that have been rotated around the longitudinal axis to best show axonal lamination. Scale bars, 20 μm (A–H) and 10 μm in (A′–D′). A, anterior; D, dorsal. (I) Quantification of axon laminar targeting behaviors in mosaically labeled RGCs (WT n = 102 axons in 50 larvae; control MO n = 94 axons in 45 larvae; *tenm3* MO n = 157 axons in 80 larvae). (J–L) Bar graphs showing the measurements for arbor thickness (J), total arbor length (K), and branching point number (L) of single RGC axons (n = 20 axons per group). All graphs show mean values ± SEM. ^∗∗∗^p < 0.001; ns, not significant by one-way ANOVA followed by Tukey’s HSD test. (M) Schematic summarizing the defects observed in the optic tecta of *tenm3* morphants. RGC axons are indicated in blue. Neuropil layers are in gray. N, neuropil. See also Figure S4.

We next examined RGC axonal arborization in the tectal neuropil. Similar to the IPL in the retina, this structure is characterized by a stereotypic lamination pattern (Xiao et al., 2011). Using the Tg(*Isl2b:Gal4;UAS:Kaede*) zebrafish line, we visualized the four main retinorecipient laminae of the tectum that, from the most superficial to the deepest, are named stratum opticum (SO), stratum fibrosum et griseum superficiale (SFGS), stratum griseum centrale (SGC), and lamina at the interface between the stratum album centrale and the stratum periventriculare (SAC/SPV; Figure 4A) (Nevin et al., 2010). In 3 dpf WT and control MO larvae, all RGC axons are restricted to these four laminae and no axons are found outside the neuropil region (Figures 4A and 4B; n = 15 larvae per group). In *tenm3* morphants, by contrast, we observed neurites projecting aberrantly into the SPV (Figures 4C and 4D, cyan arrowheads; n = 18 out of 23 larvae). 3D reconstruction and neurite tracing revealed that these processes arise principally from the deepest lamina (SAC/SPV) and, in some cases, are up to 30–40 μm long and possess several branches (Figure 4D′, cyan arrowheads). In addition, tectal laminae of *tenm3* morphants are less precisely delimited and axons aberrantly cross lamina borders (Figure 4C, yellow arrow).

To examine in more detail how the lamination defects seen at the population level arise, we labeled individual RGCs through mosaic expression of either GFP or tdTomato driven by the *ath5* promoter. As a rule, individual RGC axons arborize in a planar fashion within a single tectal lamina or sublamina (the SO and SFGS are further subdivided into 2 and 6 sublaminae, respectively) (Robles et al., 2013). This behavior was confirmed in 4 dpf control groups, where 100% of labeled axons (WT n = 102 axons in 50 larvae; control MO n = 94 axons in 45 larvae) showed planar arborization patterns (Figures 4E, 4F, 4I, and 4J; arbor thickness WT 5.1 ± 0.1 μm; control MO 5.3 ± 0.1 μm; n = 20 axons per group). In contrast, we found RGCs with abnormally laminated axonal arbors in *tenm3* morphants (Figures 4G and 4H). Intriguingly, these axons represent only a fraction of the total number of labeled RGCs (Figure 4I; 12.7%, n = 20 axons out of 157 in 80 larvae). They are characterized by possessing axonal processes projecting toward adjacent laminae (Figure 4H, cyan arrowhead) and significantly broader cross-sectional profiles (arbor thickness *tenm3* MO 16.9 ± 1.4 μm; F_2,57_ = 57.97, p < 0.0001, n = 20 axons) than those observed in control animals (Figures 4G and 4J). The total arbor length of aberrant axons is comparable to that of control groups (Figure 4K; *tenm3* MO 165.1 ± 17.4 μm; WT 173.8 ± 7.7 μm; control MO 180.5 ± 9.7 μm; F_2,57_ = 0.33, p = 0.72, n = 20 axons per group) but their number of branch points is significantly lower (Figure 4L; *tenm3* MO 5.9 ± 0.4; WT 11.5 ± 0.3; control MO 12.4 ± 0.5; F_2,57_ = 48.86, p < 0.0001, n = 60), suggesting that *tenm3* knockdown impairs their capacity to either form or stabilize new branches, without affecting overall arbor length. Taken together, these results indicate that *tenm3* is required for the correct laminar targeting and arborization of a subset of RGC axons (Figure 4M).

### Teneurin-3 Is Required for Functional Development of Orientation-Selective RGCs

**Figure 5:**
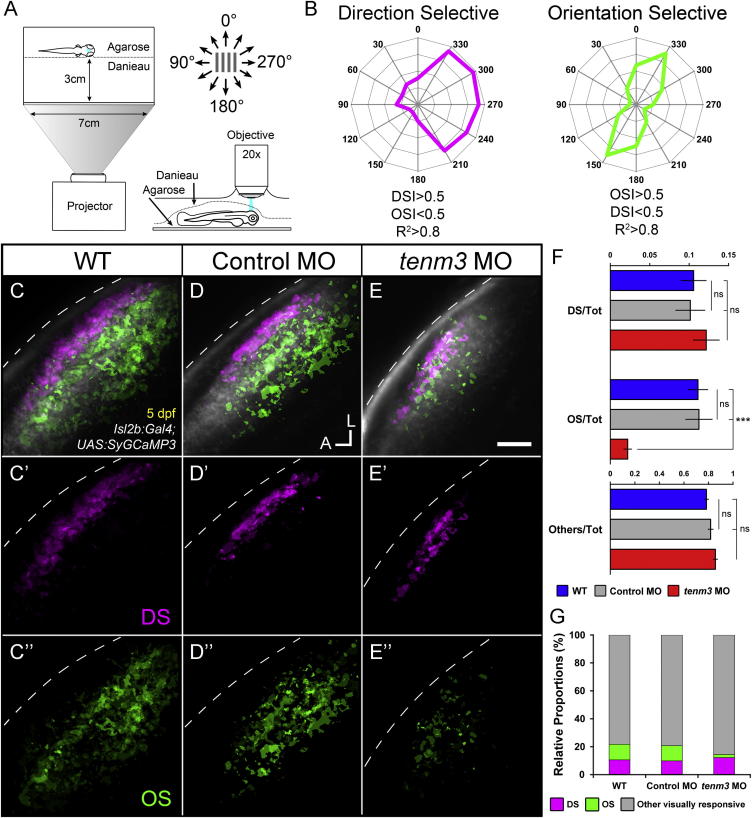
Impaired Development of Orientation-Selective RGCs Following *teneurin-3* Knockdown (A) Schematic describing the experimental setup. Larvae were immobilized in agarose and placed with one eye facing a screen, where drifting bars moving in 12 directions were projected. Visually evoked SyGCaMP3 responses were recorded in the contralateral tectal neuropil. (B) Polar plots of representative direction-selective (DS, magenta) and orientation-selective (OS, green) voxels showing relative integral responses to moving bars. Criteria employed to characterize the two classes of voxels are reported at the bottom. (C–E) Composite parametric maps across multiple 5 dpf Tg(*Isl2b:Gal4;UAS:SyGCaMP3*) larvae representing the spatial distribution of DS (magenta) and OS (green) voxels within each group (WT n = 8 larvae; control MO n = 11; *tenm3* MO n = 20). Within individual parametric maps, voxel brightness is proportional to the summed incidence of each functional response across all larvae imaged. The standard space template image derived for each group (grayscale) provides an anatomical reference. Dashed lines indicate the skin overlaying the tectum. Scale bar, 20 μm. A, anterior; L, lateral. (C′–E′) Parametric maps for DS voxels only. (C″–E″) Parametric maps for OS voxels only. (F) Bar graphs showing the ratios between defined voxel classes and total visually responsive voxels (Tot) within each group (WT n = 8 larvae; control MO n = 11; *tenm3* MO n = 20). Non-DS and non-OS voxels are classified as “others.” All graphs show mean values ± SEM. ^∗∗∗^p < 0.001; ns, not significant by one-way ANOVA followed by Tukey’s HSD test. (G) Bar graph showing the proportions of DS and OS voxel classes relative to visually responsive voxels within each group. See also Figures S2–S4 and Movies S1, S2, and S3.

To investigate the functional consequences of *tenm3* knockdown, we analyzed direction-selective (DS) and orientation-selective (OS) responses of RGC axon terminals innervating the tectal neuropil. Light or dark drifting bars moving in 12 directions were presented to one eye of 5 dpf Tg(*Isl2b:Gal4;UAS:SyGCaMP3*) transgenic larvae while functionally imaging the contralateral tectum (Figure 5A) (Nikolaou et al., 2012). Since SyGCaMP3 is based on the fusion between the synaptic vesicle protein synaptophysin and the genetically encoded calcium indicator GCaMP3, this transgenic line enables the targeting of the probe specifically to RGC presynaptic terminals and hence the functional analysis of RGCs within the tectal target. RGCs of all three animal groups respond to drifting bars (Movies S1, S2, and S3) and exhibit complex patterns of stimulus responses (Figure S2). In order to characterize and map visual response properties (i.e., direction and orientation selectivity) present in the retinal input to the tectum, we used a voxel-wise analysis strategy that is independent of cellular and neuropil morphology (Nikolaou et al., 2012). Only visually responsive voxels were subjected to further characterization. Direction- and orientation-selective indices (DSI and OSI) based on fitted von Mises profiles were calculated together with an estimate for their goodness of fit, R^2^ (Lowe et al., 2013) (see Supplemental Experimental Procedures). For a voxel to be regarded as DS or OS, mutually exclusive criteria were employed: DS if R^2^ > 0.8, DSI > 0.5, and OSI < 0.5; and OS if R^2^ > 0.8, OSI > 0.5, and DSI < 0.5 (Figure 5B). Functional maps in which DS and OS voxels are color-coded, obtained from individual larvae, were spatially coregistered to generate parametric composite maps (Figures 5C–5E; WT n = 8 larvae; control MO n = 11; *tenm3* MO n = 20). Analyzing the DS RGC input to the tectum, we observed that in all three experimental groups DS responses are present (Figures 5C′–5E′). Moreover, the normal laminar organization of DS voxels within the superficial region of SFGS (Nikolaou et al., 2012) is preserved in *tenm3* morphants (Figures 5C′–5E′). Further analysis of DS RGC subtypes revealed that all three DS RGC populations—tuned to anterior (∼260°), dorsoposterior (∼40°), and ventroposterior (∼150°) motion—found in control groups (Nikolaou et al., 2012) are also present in *tenm3* morphants (Figure S3). Overall, no difference between *tenm3* morphants and control groups was observed in the DS RGC input to the tectum.

In contrast, we found that the OS RGC input to the tectum is severely impaired upon *tenm3* knockdown. Specifically, the overall number of OS voxels is decreased in *tenm3* morphants (Figures 5C″–5E″). In addition, the OS RGC voxels that are typically found in deeper sublaminae of SFGS with little or no overlap with DS RGCs in control animals (Figures 5C and 5D) (Nikolaou et al., 2012) show a substantial degree of overlap with DS voxels in *tenm3* morphants (Figure 5E). To further confirm the OS RGC impairment, we analyzed the relative proportions of functional response classes within each experimental group. In *tenm3* morphants, we found a significant decrease in the ratio between OS voxels and the total population of visually responsive voxels (Figure 5F; OS/tot *tenm3* MO 0.022 ± 0.004, n = 20 larvae; WT 0.111 ± 0.012, n = 8; control MO 0.112 ± 0.016, n = 11; F_2,36_ = 24.61, p < 0.0001), so the OS input becomes the smallest population of RGCs responding to drifting bars in this group (Figure 5G). The relative proportions of DS and non-DS/non-OS (classified as “others”) voxel populations, however, were similar among the three animal groups (Figures 5F and 5G; DS/tot WT 0.105 ± 0.015, control MO 0.101 ± 0.018, *tenm3* MO 0.121 ± 0.016, F_2,36_ = 0.42, p = 0.66; others/tot WT 0.783 ± 0.016, control MO 0.817 ± 0.019, *tenm3* MO 0.856 ± 0.016, F_2,36_ = 3.06, p = 0.059, n = 39 larvae), suggesting no impairment by *tenm3* knockdown. These functional results indicate that visual responses of OS RGCs are affected by *tenm3* knockdown whereas DS RGCs develop normally, therefore reinforcing the possible role of *tenm3* in the assembly of specific visual circuits. All structural and functional phenotypes observed using *tenm3* MO were confirmed in larvae injected with a second splice-blocking MO against *tenm3* (*tenm3* MO 2; Figure S4), supporting the specificity of gene knockdown.

## Discussion

Recent studies in *Drosophila* showed that teneurins are involved in establishing specific synaptic circuits (Hong et al., 2012; Mosca et al., 2012). However, a similar role in vertebrate neural circuit wiring has not yet been demonstrated. Here, we report that Teneurin-3 is required for the correct structural and functional development of RGCs in zebrafish. RGCs and their pre- and postsynaptic cellular targets (i.e., amacrine cells and tectal neurons, respectively) express *tenm3* during the period of intense synapse formation (2–5 dpf), suggesting an instructive role in synaptic matching through homophilic interactions between neuronal partners along the visual pathway. *Tenm3* knockdown produces laminar targeting errors of RGC dendrites and axons, indicating that Tenm3 acts in both the IPL of the retina and the tectal neuropil. Intriguingly, these errors appear to be restricted to a subset of RGCs, hinting that Tenm3 acts in specific RGC subtypes and that Tenm3-negative cells are unaffected. Consistent with this hypothesis, when we examined the functional development of visual response properties conveyed by RGCs, we observed that the OS retinal input to the tectum is strongly impaired whereas direction selectivity is not affected in *tenm3* morphant larvae. This does not exclude, however, that additional RGC functional subtypes may be affected in *tenm3* morphants. Previous studies in mice showed that Teneurin-3 regulates the development of topography in the retinogeniculate (Leamey et al., 2007) and retinocollicular pathways (Dharmaratne et al., 2012), specifically for the ipsilaterally projecting RGC population. However, the fact that *teneurin-3* is not exclusively expressed in ipsilaterally projecting RGCs (Leamey et al., 2007) and is also found in the visual system of species where RGCs project contralaterally only, like chick (Kenzelmann-Broz et al., 2010) and zebrafish (Mieda et al., 1999; this study), clearly suggests additional functions in vertebrate visual system development.

Taken together, our findings support a role for Tenm3 in the establishment of functional cell subtype-specific wiring in vertebrates. What developmental mechanisms does Tenm3 regulate? It is generally accepted that molecules mediating homophilic cell-cell adhesion instruct the recognition between pre- and postsynaptic elements by triggering specific synapse formation/stabilization (Sanes and Yamagata, 2009; Williams et al., 2010). In addition, teneurin-mediated homophilic recognition and subsequent formation of cell-adhesion partners leads to inhibition of neurite outgrowth (Beckmann et al., 2013). Thus, the simplest hypothesis is that *tenm3* (by being expressed in RGCs, amacrine cells, and tectal neurons) controls the lamination of RGC neurites through stabilization of branches contacting neurites of *tenm3*-expressing cells. Homophilic adhesion has been extensively studied in the IPL of the chick retina, where different immunoglobulin superfamily adhesion molecules are expressed by specific subsets of cells and control the precise sublaminar matching of their neurites (Yamagata and Sanes, 2008, 2012). Interestingly, this matching mechanism appears to be conserved in higher visual targets. For example, evidence in mouse showed that Cadherin-6 mediates the axon-target recognition between a specific subset of RGCs and their target nuclei in the brain (Osterhout et al., 2011). An alternative mechanism that might regulate RGC neurite arborization is the neurite costratification between morphologically and functionally related cells expressing the same combination of adhesive proteins. This kind of interaction certainly occurs during IPL development. In studies where single or multiple retinal cell classes were selectively eliminated, the remaining cellular components could form a stratified IPL, therefore suggesting that no single pre- or postsynaptic retinal cell class is strictly essential for IPL formation (Kay et al., 2004; Randlett et al., 2013). Further experiments are needed to determine the exact mechanisms of action of Tenm3 and in which cell subtypes it is expressed. Meanwhile, our results presented here point toward an important role for teneurins in the development of vertebrate neural circuit specificity.

## Experimental Procedures

### Transgenic Lines and Constructs

Transgenic lines Tg(*Isl2b:Gal4*) and Tg(*UAS:SyGCaMP3*) have been described previously (Ben Fredj et al., 2010; Nikolaou et al., 2012). Transgenic line Tg(*UAS:Kaede*) was a gift of Prof. Chi Bin-Chien. The *UAS:GFP* and *UAS:tdTomato* DNA constructs were described previously (Ben Fredj et al., 2010), and the *Ath5:Gal4* plasmid was a gift of Prof. Steve Wilson (UCL, UK). All animal procedures were approved by the local Animal Welfare and Ethics Review Body (King’s College London) and were carried out in accordance with the Animals (Scientific Procedures) Act 1986, under license from the United Kingdom Home Office.

### Functional Imaging

Confocal imaging was performed using an LSM 710 confocal microscope equipped with a spectral detection scan head and a 20×/1.0 NA water-immersion objective (Carl Zeiss). Functional time series of visually evoked SyGCaMP3 responses were acquired at a rate of 4.1 Hz and 0.415 × 0.415 μm resolution (256 × 256 pixels) and 1 AU pinhole aperture. Visual stimulation and voxel-wise analysis of functional data were performed as described previously (Nikolaou et al., 2012) (see Supplemental Experimental Procedures).

### Statistical Analyses

The statistical significance of the differences between mean values and in the proportion of diffuse RGCs among groups was determined by one-way ANOVA followed by Tukey’s HSD test and chi-square test, respectively, using SigmaPlot (Systat Software). The criterion for statistical significance was set at p < 0.05 and results are represented as mean ± SEM.

## Supplemental Information

Document S1. Supplemental Experimental Procedures and Figures S1–S4

Movie S1. Functional RGC Responses to Drifting Bars in a Representative 5 dpf WT Zebrafish Larva, Related to Figures 5 and S2 Time-lapse functional data of a representative 5 dpf WT larva. The movie encompasses an entire tuning experiment in which all 12 directions of bar motion plus a blank screen null stimulus are presented to one eye of the immobilized zebrafish larva. Unprocessed SyGCaMP3 responses are shown on the left, whereas ΔF/F responses are reported on the right. Time given in min:sec. The same data are presented as parametric map and montage in Figures S2A and S2D, respectively.

Movie S2. Functional RGC Responses to Drifting Bars in a Representative 5 dpf Control MO-Injected Zebrafish Larva, Related to Figures 5 and S2 Time-lapse functional data of a representative 5 dpf control MO-injected larva. The movie encompasses an entire tuning experiment in which all 12 directions of bar motion plus a blank screen null stimulus are presented to one eye of the immobilized zebrafish larva. Unprocessed SyGCaMP3 responses are shown on the left, whereas ΔF/F responses are reported on the right. Time given in min:sec. The same data are presented as parametric map and montage in Figures S2B and S2E, respectively.

Movie S3. Functional RGC Responses to Drifting Bars in a Representative 5 dpf Teneurin-3 Morphant Zebrafish Larva, Related to Figures 5 and S2 Time-lapse functional data of a representative 5 dpf *tenm3* morphant larva. The movie encompasses an entire tuning experiment in which all 12 directions of bar motion plus a blank screen null stimulus are presented to one eye of the immobilized zebrafish larva. Unprocessed SyGCaMP3 responses are shown on the left, whereas ΔF/F responses are reported on the right. Time given in min:sec. The same data are presented as parametric map and montage in Figures S2C and S2F, respectively.

Document S2. Article plus Supplemental Information
