# Supplementary material for: Teneurin-3 Specifies Morphological and Functional Connectivity of Retinal Ganglion Cells in the Vertebrate Visual System
Source: Cell Rep. 2013 Oct 31;5(3):582–92. doi: 10.1016/j.celrep.2013.09.045 (PMC3898612; doi:10.1016/j.celrep.2013.09.045)
Supplement: Document S1. Supplemental Experimental Procedures and Figures S1–S4 [file mmc1.pdf]

Supplemental Information

Teneurin-3 Specifies Morphological and Functional Connectivity of Retinal Ganglion Cells in the Vertebrate Visual System

Paride Antinucci,[1] Nikolas Nikolaou,[1] Martin P Meyer,[1] and Robert Hindges[1,]*

[1] MRC Centre for Developmental Neurobiology,
King's College London, Guy's Campus,
London SE1 1UL, UK

*Correspondence: robert.hindges@kcl.ac.uk

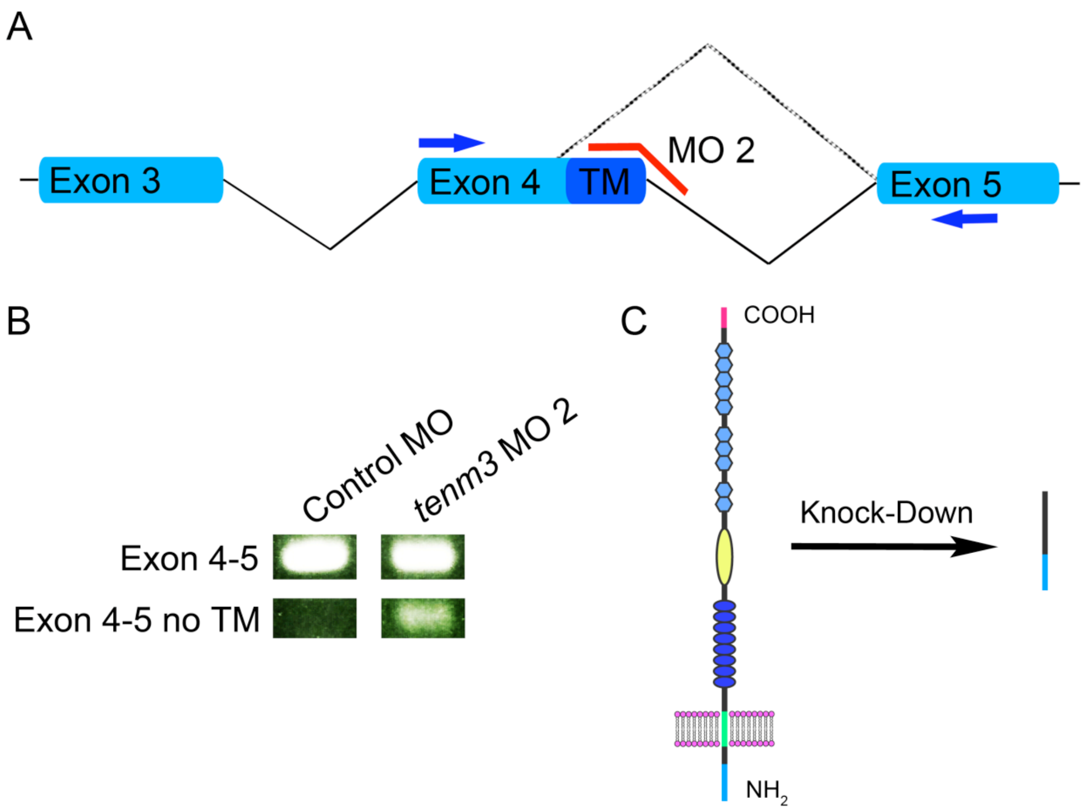

Figure S1. Design and Effect of the Second Splice-Blocking Morpholino Against *teneurin-3*, Related to Figure 1 and S4

(A) Schematic detailing the targeting site of the second splice-blocking *tenm3* morpholino (MO 2), which is shown in red. Importantly, this MO targets a non-overlapping region of *tenm3* mRNA, namely the boundary between exon 4 and intron 4. Exons are represented in cyan. Solid lines indicate introns. The dashed line indicates deletion from exon 4 of the sequence encoding the transmembrane domain (TM, blue) caused by the activation of a cryptic splice donor site following *tenm3* MO 2 injections. Primers used for RT-PCR (B) are reported as blue arrows.

(B) RT-PCR analysis of *tenm3* mRNA structure in control MO- and *tenm3* MO 2-injected embryos. A shorter splice variant is present in *tenm3* morphants. cDNA sequence comparison after DNA sequencing revealed that the short splice variant lacks 64 bp from the 3' region of exon 4. Importantly, this region encodes the transmembrane domain. This deletion leads to a frameshift in exon 5 and a consequent early stop codon in the same exon, resulting in deletion of the transmembrane and extracellular domains.

(C) Schematic detailing the effect of *tenm3* MO 2, which produces the deletion of Tenm3 transmembrane and extracellular domains. The full-length protein is represented on the left. The N-terminus is located intracellularly, whereas the C-terminus is in the extracellular space.

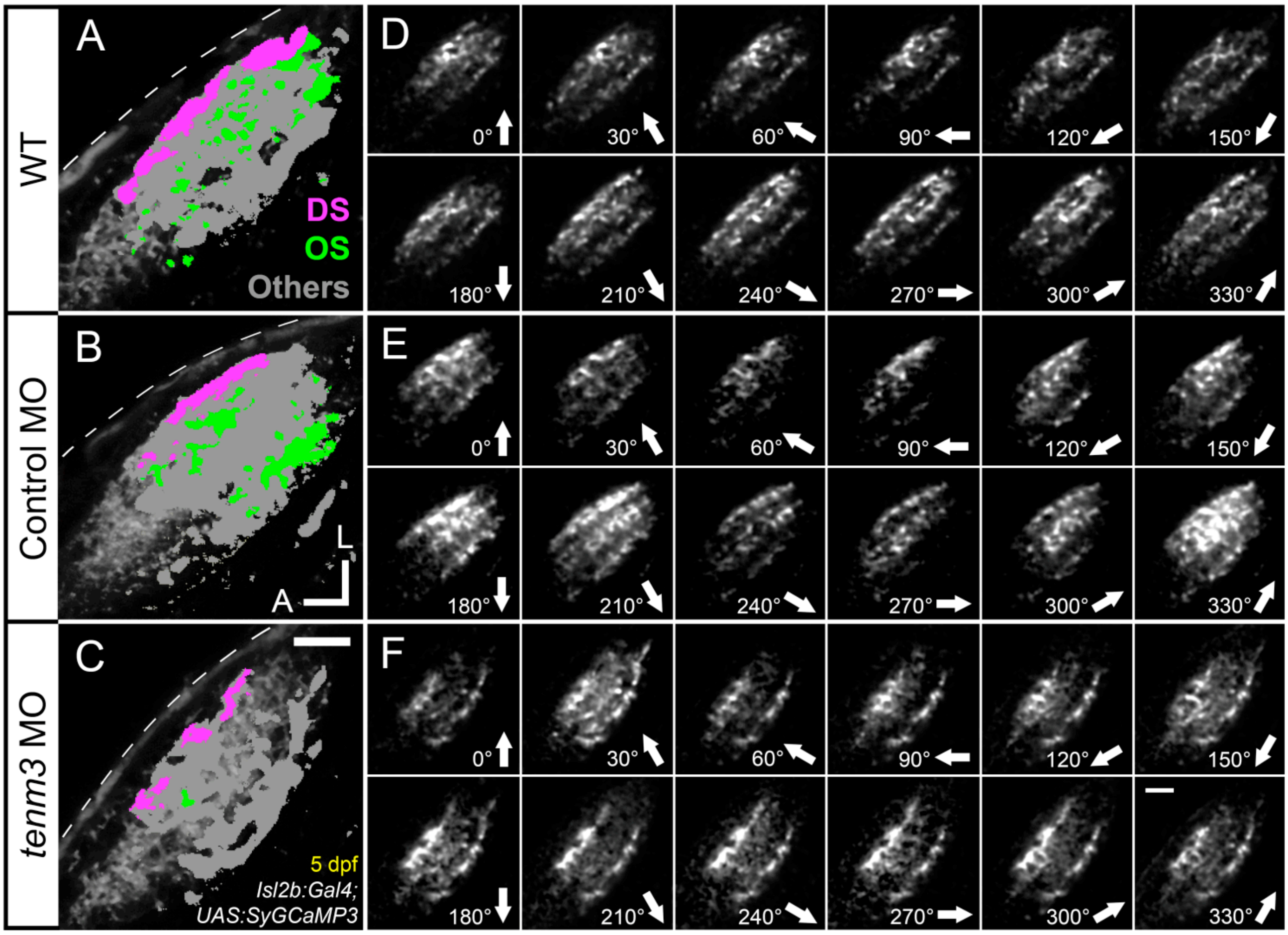

Figure S2. RGC Functional Responses to Drifting Bars, Related to Figure 5 and Movies S1-S3

(A-C) Parametric maps of single 5 dpf Tg(*Isl2b:Gal4;UAS:SyGCaMP3*) larvae (one larva per animal group) representing the spatial distribution of direction-selective (DS, magenta), orientation-selective (OS, green) and other visually responsive voxels (others, grey) superimposed onto the mean fluorescence images of SyGCaMP3-expressing axons (greyscale). Note that the *tenm3* morphant larva (C) shows substantially less OS voxels than WT and control MO-injected larvae (A and B). Dashed lines indicate the skin overlaying the tectum.

(D-E) Montages showing integral responses (grayscale) of all voxels (RGC axons expressing SyGCaMP3) in the tectal neuropil of the larvae described in (A-C). Note that all three larvae exhibit complex response patterns to moving bars. Direction of motion is shown on the bottom right in each panel. Scale bars = 20 μm. A, anterior; L, lateral. The three representative larvae shown in (A-E) are the same used to generate Movies S1-S3.

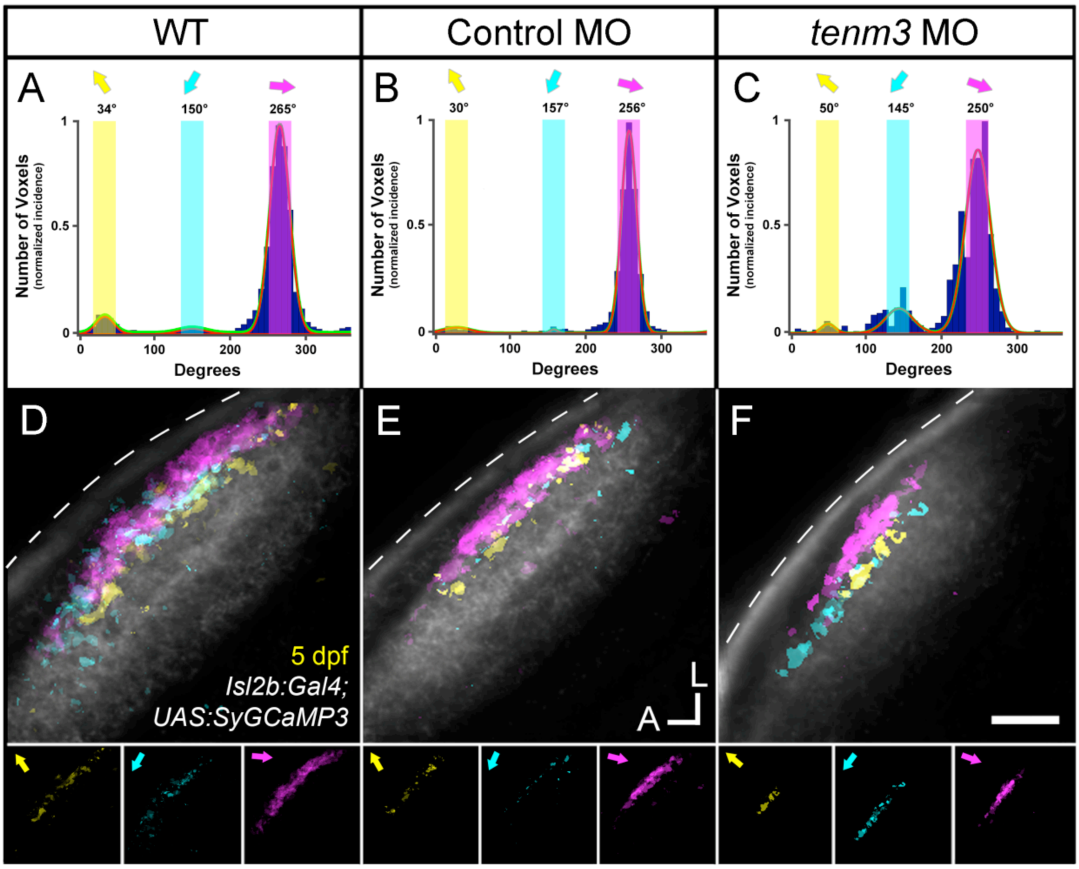

Figure S3. The Three Direction-Selective RGC Subtypes Are Not Affected by *teneurin-3* Knock-Down, Related to Figure 5

(A-C) Cumulative histograms summarizing the incidence of direction-selective (DS) voxels within each group (WT n = 8 larvae; control MO n = 11; *tenm3* MO n = 20). Fitted von-Mises distributions reveal three populations of DS voxels tuned to three different directions of motion. Individual peak preferred angles are reported above. Note that all three animal groups develop three DS RGC subtypes. Moreover, the sizes of individual DS voxel populations are comparable across groups, with the dominant input corresponding to anterior motion selectivity.

(D-F) Composite parametric maps across multiple 5 dpf larvae representing the spatial distribution of the three DS voxel subtypes within each group (WT n = 8 larvae; control MO n = 11; *tenm3* MO n = 20). Note that the laminar organization of the three DS voxel subtypes in the tectal neuropil is analogous in all three groups, namely they are confined to superficial layers of the SFGS. Individual parametric maps for each voxel subtype are shown at the bottom. Color coding as per (A-C). The standard space template image derived for each group (greyscale) provides an anatomical reference. Dashed lines indicate the skin overlaying the tectum. Scale bar = 20 μm. A, anterior; L, lateral.

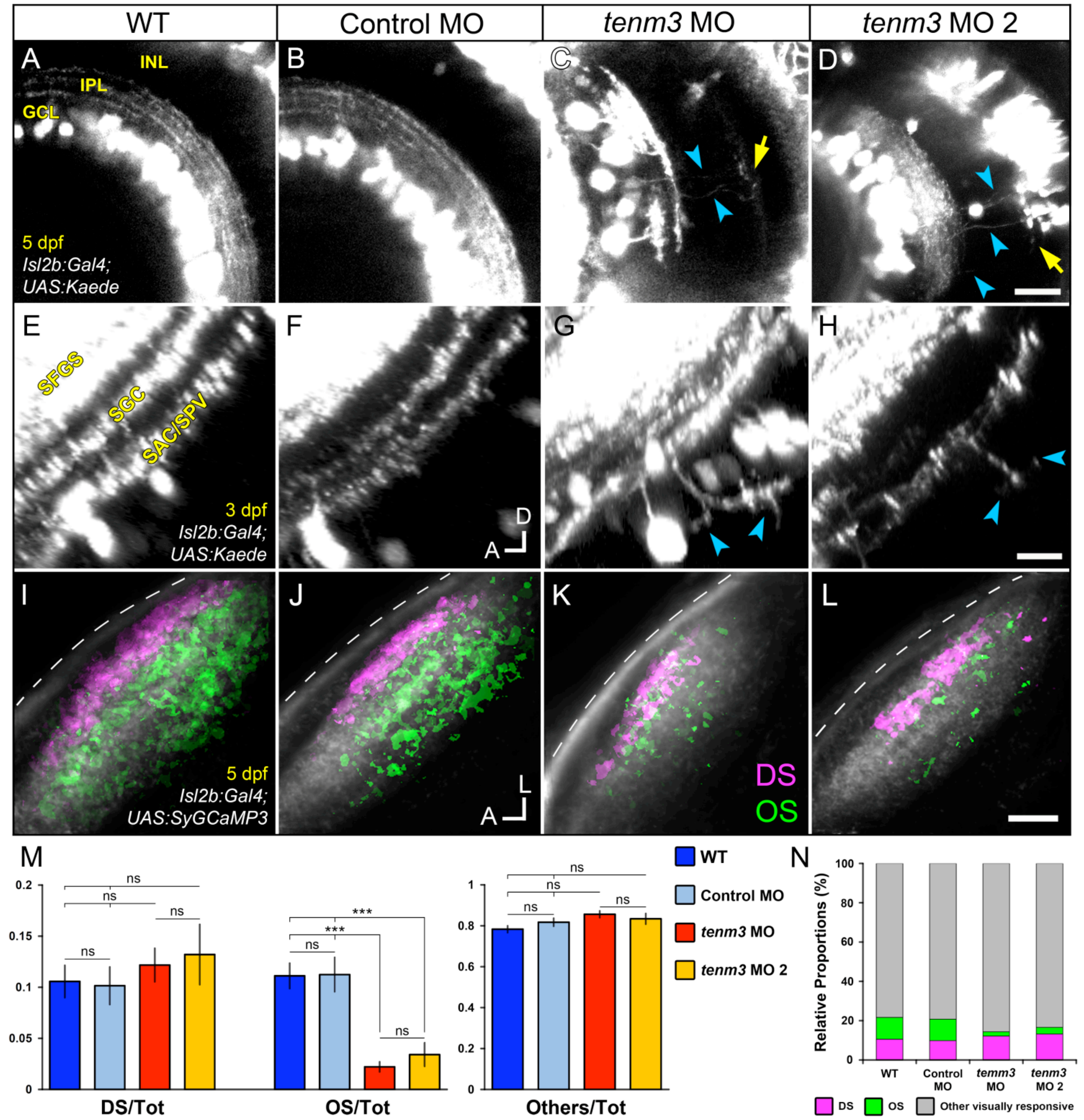

Figure S4. Two Different Morpholino Oligonucleotides Against *teneurin-3* Produce Equivalent Structural and Functional Results, Related to Figure 2, 4, 5 and S1

(A-D) Kaede-expressing RGCs in the retina of 5 dpf WT, control MO-, *tenm3* MO-, and *tenm3* MO 2-injected Tg(*Isl2b:Gal4;UAS:Kaede*) zebrafish larvae. Aberrant RGC neurite extensions in the inner nuclear layer (INL) are detected in *tenm3* MO-injected larvae (C) as well as in *tenm3* MO 2-injected larvae (D, cyan arrowheads) but not in WT (A) and Control MO-injected (B) larvae. In some cases these processes reach the outer plexiform layer both in *tenm3* MO- (C) and *tenm3* MO 2-injected larvae (D, yellow arrows) but not in control larvae. All images represent maximum intensity projections of ~20 μm confocal Z stacks. Scale bar = 20 μm. GCL, ganglion cell layer; IPL, inner plexiform layer. WT, control MO and *tenm3* MO images in Figure 2 are reported here for comparison.

(E-H) RGC axon lamination in deep laminae of the tectal neuropil of 3 dpf Tg(*Isl2b:Gal4;UAS:Kaede*) zebrafish larvae. Aberrant extensions projecting into the stratum periventriculare (SPV) are detected in *tenm3* MO-injected larvae (G) as well as in *tenm3* MO 2-injected larvae (H, cyan arrowheads) but not in control larvae (E and F). All images represent maximum intensity projections of ~50 µm confocal Z stacks that have been rotated around the longitudinal axis to best show axonal lamination. Scale bar = 10 µm. A, anterior; D, dorsal. SFGS, stratum fibrosum et griseum superficiale; SAC, stratum album centrale. WT, control MO and *tenm3* MO images in Figure 4 are reported here for comparison.
(I-L) Composite parametric maps across multiple 5 dpf Tg(*Isl2b:Gal4;UAS:SyGCaMP3*) larvae representing the spatial distribution of direction-selective (DS, magenta) and orientation-selective (OS, green) voxels within each group (WT n = 8 larvae; control MO n = 11; *tenm3* MO n = 20; *tenm3* MO 2 n = 8). Note that both in *tenm3* MO- (K) and *tenm3* MO 2-injected larvae (L) the overall number of OS voxels is decreased compared to control larvae (I and J). Within individual parametric maps, voxel brightness is proportional to the summed incidence of each functional response across all larvae imaged. The standard space template image derived for each group (greyscale) provides an anatomical reference. Dashed lines indicate the skin overlaying the tectum. Scale bar = 20 µm. A, anterior; L, lateral. WT, control MO and *tenm3* MO images in Figure 5 are reported here for comparison.
(M) Ratios between defined voxel classes and total visually responsive voxels within each group. Both in *tenm3* MO- and *tenm3* MO 2-injected larvae there is a significant change in the ratio between OS voxels and the total population of visually responsive voxels (OS/tot *tenm3* MO 0.022 ± 0.004, n = 20 larvae; *tenm3* MO 2 0.034 ± 0.011, n = 8; WT 0.111 ± 0.012, n = 8; control MO 0.112 ± 0.016, n = 11; $F_{(3,44)} = 18.88$, $p < 0.0001$). No significant change was observed in the relative proportions of the other voxel populations (DS/tot WT 0.105 ± 0.015, control MO 0.101 ± 0.018, *tenm3* MO 0.121 ± 0.016, *tenm3* MO 2 0.132 ± 0.029, $F_{(3,44)} = 0.37$, $p = 0.775$; Others/tot WT 0.783 ± 0.016, control MO 0.817 ± 0.019, *tenm3* MO 0.856 ± 0.016, *tenm3* MO 2 0.833 ± 0.025, $F_{(3,44)} = 1.999$, $p = 0.128$, n = 47 larvae). Non-DS and non-OS voxels are classified as ‘others’. All graphs show mean values ± SEM. ***p < 0.001; ns, not significant by one-way ANOVA followed by Tukey’s HSD test.
(N) Bar graph showing the proportions of DS and OS voxel classes relative to visually responsive voxels within each group. Note that the OS input becomes the smallest population of RGCs responding to drifting bars both in *tenm3* MO- and *tenm3* MO 2-injected larvae.

SUPPLEMENTAL MOVIE TITLES AND LEGENDS

Movie S1. Responses of SyGCaMP3-Expressing Axons in the Tectal Neuropil of a WT Larva Evoked by Drifting Bars, Related to Figure 5 and S2

Time-lapse functional data of a representative 5 dpf WT larva. The movie encompasses an entire tuning experiment in which all 12 directions of bar motion plus a blank screen null stimulus are presented to one eye of the immobilized zebrafish larva. Unprocessed SyGCaMP3 responses are shown on the left, whereas ΔF/F responses are reported on the right. Time given in min:sec. The same data are presented as parametric map and montage in Figures S2A and S2D, respectively.

Movie S2. Responses of SyGCaMP3-Expressing Axons in the Tectal Neuropil of a Control MO-Injected Larva Evoked by Drifting Bars, Related to Figure 5 and S2

Time-lapse functional data of a representative 5 dpf control MO-injected larva. The movie encompasses an entire tuning experiment in which all 12 directions of bar motion plus a blank screen null stimulus are presented to one eye of the immobilized zebrafish larva. Unprocessed SyGCaMP3 responses are shown on the left, whereas ΔF/F responses are reported on the right. Time given in min:sec. The same data are presented as parametric map and montage in Figures S2B and S2E, respectively.

Movie S3. Responses of SyGCaMP3-Expressing Axons in the Tectal Neuropil of a *teneurin-3* Morphant Larva Evoked by Drifting Bars, Related to Figure 5 and S2

Time-lapse functional data of a representative 5 dpf *tenm3* morphant larva. The movie encompasses an entire tuning experiment in which all 12 directions of bar motion plus a blank screen null stimulus are presented to one eye of the immobilized zebrafish larva. Unprocessed SyGCaMP3 responses are shown on the left, whereas ΔF/F responses are reported on the right. Time given in min:sec. The same data are presented as parametric map and montage in Figures S2C and S2F, respectively.

EXTENDED EXPERIMENTAL PROCEDURES

Animals

Zebrafish were maintained at 28.5°C on a 14 hr ON/10 hr OFF light cycle in Danieau solution [58 mM NaCl, 0.7 mM KCl, 0.4 mM $MgSO_4$, 0.6 mM $Ca(NO_3)_2$ 5.0 mM HEPES, pH 7.6]. The AB strain of zebrafish was used for *in situ* hybridization, RT-PCR, visual background adaptation and mosaic labeling of retinal ganglion cells (RGCs). Transgenic lines used in this study include Tg(*Isl2b:Gal4*) (Ben Fredj et al., 2010), Tg(*UAS:Kaede*) (gift of Prof. Chi Bin-Chien) and Tg(*UAS:SyGCaMP3*) (Nikolaou et al., 2012). Functional imaging experiments were performed in the pigmentation mutant *nacre,* which lacks all neural crest derived melanophores (Lister et al., 1999). Larvae used for all the other experiments were raised in 0.003% phenylthiourea (Sigma) in 1x Danieau solution to avoid pigment formation. This work was approved by the local Animal Care and Use Committee (King's College London), and was carried out in accordance with the Animals (Experimental Procedures) Act, 1986, under licence from the United Kingdom Home Office.

In Situ Hybridization

To make the *tenm3* antisense riboprobe, a 981 bp cDNA fragment (ORF 7034-8014) was cloned into a StrataClone Blunt PCR Cloning Vector pSC-B-amp/kan (Agilent Technologies), and the orientation of the insertion was determined by DNA sequencing. The sequences of primers used to amplify the fragment through PCR are as follows: forward primer 5'-GGGACTATGACATTCAAG CAGGTC-3'; reverse primer 5'-CATTGTTGGCACTGTCGGCCAG-3'. The antisense RNA probe was generated from the linearized plasmid using T3 RNA polymerase (Life Technologies) and digoxigenin-labeled nucleotides (Roche). Whereas, the sense digoxigenin-labeled RNA probe was generated using T7 RNA polymerase (Life Technologies). After synthesis, riboprobes were treated with DNase I (Ambion) for 15 minutes at 37°C.

The same protocol described in Thisse and Thisse (2008) was used to perform whole-mount *in situ* hybridisations. Briefly, embryos were fixed in 4% paraformaldehyde (PFA) in PBS overnight at 4°C, dehydrated in methanol and stored at -20°C. Subsequently, embryos were rehydrated in 75% methanol in PBT (0.1% Tween 20 in PBS), 50% methanol in PBT, 25% methanol in PBT, and PBT for 5 minutes each. 2, 3 and 5 dpf embryos were digested with proteinase K (10 µg/ml; Sigma) at room temperature for 20, 30 and 60 minutes, respectively. Subsequently, they were fixed in 4% PFA in PBS for 20 minutes at room temperature and washed several times in PBT. They were then transferred to hybridisation mix (HM; 50% formamide, 5x SSC, 0.1% Tween 20, 50 µg/ml heparin, 0.5 mg/ml yeast tRNA, and 9 mM citric acid to pH 6.0 in DEPC ddH_2O) and incubated for 4 hours at 65°C. The HM was replaced with a solution containing 1 µg/ml of digoxigenin-labeled RNA probe in HM and the embryos were incubated overnight at 65°C. Washes were performed at the hybridisation temperature in 65% HM/35% 2x SSC, 35% HM/65% 2x SSC, 2x SSC, for 10 minutes each and, finally, in 0.2x SSC + 0.1% Tween 20 for 20 minutes and 2 times 20 minutes each in 0.1x SSC + 0.1% Tween 20. A series of washes were performed at room temperature in 65% 0.2x SSC/35% PBT, 35% 0.2x SSC/65% PBT, and PBT for 10 minutes each. Embryos were then incubated in blocking solution (5% sheep serum in PBT) for 4 hours at room temperature. The blocking solution was replaced with alkaline phosphatase-conjugated anti-digoxigenin Fab fragments (Roche) diluted 1:2000 in blocking solution and embryos were incubated at 4°C overnight. After washing at least 8 times 1 hour each in PBT at room temperature followed by

overnight wash in PBT at 4°C, embryos were rinsed 3 times 20 minutes each in NTMT staining buffer (0.1 M Tris-HCl pH 9.5, 50 mM $MgCl_2$, 0.1 M NaCl, 0.1% Tween 20). The staining buffer was then replaced with 2% NBT/BCIP stock solution (Roche) in NTMT and embryos were incubated in the dark at room temperature. To stop the reaction, embryos were washed in PBS and fixed in 4% PFA in PBS for 20 minutes at room temperature. After several washes in PBS, embryos were washed in 15% and 30% sucrose in PBS for 2 hours each at room temperature, then in 40% sucrose in PBS overnight at 4°C. Subsequently, embryos were embedded in OCT (VWR International) and frozen in dry ice. Finally, 20 µm-thick sections were cut using a cryostat and stored in 70% glycerol in PBS.

Morpholino and DNA Microinjections

2-2.5 ng/1.8 nl (0.24-0.3 pmols) of morpholino oligonucleotides (MOs; Gene Tools) in Danieau solution were injected into one-cell stage zebrafish embryos. The sequences of MOs used are as follows: splice-blocking *tenm3* MO 5'-ACGGTTGCTctgtggaaaaaaatca-3' (intronic sequence in lower case); splice-blocking *tenm3* MO 2 5'-taggcagtgttaaacttacCAATGC-3'; standard control MO 5'-CCTCTTACCTCAGTTACAATTTATA-3'.

In order to mosaically label RGCs, an activator plasmid containing Gal4 driven by an upstream *ath5* promoter (*Ath5:Gal4*) (gift of Prof. Steve Wilson, UCL, UK) was co-injected with effector plasmids, where expression is driven by a UAS motif in frame with either GFP (*UAS:GFP*) or tdTomato (*UAS:tdTomato*) (Ben Fredj et al., 2010). The plasmids were injected at a concentration of 20 ng/µl each in Danieau solution. Plasmid DNA was prepared using miniprep kits (Qiagen).

RT-PCR

Total RNA was isolated from multiple dechorionated embryos (~30) using TRIzol reagent (Life Technologies). cDNA was synthesized using SuperScript III reverse transcriptase (Life Technologies) and random primers (Promega, C1181). The obtained cDNA was then used as template for amplification through PCR. For *tenm3* MO, the sequences of the primers are as follows: forward primer 5'-ATGCCATCCTCTCTCTCCAGTCCA-3'; reverse primer 5'-ACTTC TTGAACTTGAAGGCGCTGC-3'. They target exon 2 and exon 4 of the tenm3-001 splice variant, respectively. Whereas, for *tenm3* MO2 the sequences of the primers are as follows: forward primer 5'-CGGCCACGCCAGGCTACACTATG-3'; reverse primer 5'-TCCTGCAGCTGCCAATTCAGTCC-3'. They target exon 4 and exon 5 of the tenm3-001 splice variant, respectively. Full-length and shorter splice variants were extracted from agarose gel using QIAquick Gel Extraction Kits (Qiagen), cloned into StrataClone Blunt PCR Cloning Vectors pSC-B-amp/kan (Agilent Technologies) and verified by sequencing.

Visual Background Adaptation

Visual background adaptation of 4 dpf larvae was assayed by exposure to bright light for more than 3 hours, followed by visual inspection of pigmentation using a stereomicroscope.

Imaging

Imaging was performed using an LSM 710 confocal microscope equipped with a spectral detection scan head and a 20x/1.0 NA water-immersion objective (Carl Zeiss). Functional time-series of visually-evoked SyGCaMP3 responses were acquired at a rate of 4.1 Hz and 0.415x0.415 µm resolution (256x256 pixels) and 1 AU pinhole aperture. The average diameter of a presynaptic bouton in zebrafish RGCs is ~0.8 µm (Meyer and Smith, 2006). Thus, the physical X-Y dimensions of voxels are below that of a typical presynaptic bouton. Excitation was provided by 488 nm multi-

line laser. Optical sections were obtained at <1.6 µm intervals, and maximum intensity projections of Kaede-, GFP- and tdTomato-positive neurons were generated using either ImageJ (NIH) or ZEN (Carl Zeiss). Imaging of cryosections after *in situ* hybridizations was performed using a Zeiss Axioskop microscope connected to a cooled monochrome CCD camera (Retiga EXi Blue) and Volocity acquisition software (PerkinElmer).

Data Analysis

All images were processed using either ImageJ (NIH) or ZEN (Carl Zeiss). To make Figure 2E, *a* rectangular region of interest was drawn across a short, relatively straight stretch of the IPL with pronounced dendrite stratification. The Plot Profile function in ImageJ (NIH) was applied to the rectangle to calculate the fluorescence intensity trace across the IPL width. The values obtained from multiple larvae were then normalized and averaged using SigmaPlot (Systat Software). Analyses of IPL width and RGC axon thickness, length and branching were carried out using ImageJ (NIH).

Visual Stimulation and Voxel-Wise Analysis

Visual stimulation was performed as previously described (Nikolaou et al., 2012). In summary, larvae with SyGCaMP3 expression in RGC presynaptic terminals, were restrained in 2% low melting point agarose (Sigma) in 1x Danieau solution, mounted with the dorsal side up onto a customized glass platform and placed in a Danieau-filled chamber. The agarose was sufficient to restrain the larvae so that anesthesia was not required. The agarose surrounding one eye was removed allowing an unobstructed view of the projected image on one side of the chamber, which served as a projection screen. Time-series were recorded from the contralateral tectal neuropil. The projected image filled a visual field of approximately 97° by 63°. Visual stimuli consisted of light (56 cd/m^2) or dark bars (8 cd/m^2) (175% and 25% of mean, respectively) on a mean gray background (32 cd/m^2). Since no qualitative differences between light and dark bars responses were noted, data for the two stimuli were combined. Each bar was 10° in width moving at 20°/s and separated from the preceding bar by 30°, therefore enabling more than one bar on the screen at any one time. The long axis of the bar was orthogonal to the direction of motion. Bars were presented at 12 different directions evenly spaced across 360° and displayed in a pseudo-random order. A blank screen null condition of 2 s was also interleaved. Each inter-epoch interval was 8 s to enable the SyGCaMP3 signal to return baseline. Visual experiments were generated and controlled using custom written Labview and Matlab code (MathWorks) implemented on a ViSaGe stimulus presenter (Cambridge Research Systems, UK) and delivered via a DLP picoprojector (Optoma).

Voxel-wise analysis of direction- and orientation-selective responses and generation of composite maps were performed as previously described (Nikolaou et al., 2012) excepting that a goodness of fit (R^2) was used in the identification of functional classes of voxels (Lowe et al., 2013). Briefly, a threshold for each voxel within an acquisition image was determined from the variance of ΔF changes during the inter-epoch intervals and null condition – threshold ≡ 5 standard deviations. All voxels that were supra-threshold within at least two visually presentation epochs were regarded as visually responsive and subjected to further characterization: direction selectivity and orientation selectivity. Direction- and orientation-selective indices (DSI and OSI) (Niell and Stryker, 2008) based on fitted von-Mises profiles (Swindale, 1998) were calculated together with an estimate for their goodness of fit – R^2 (Lowe et al., 2013). To minimize cross-talk and over-fitting associated with DSI and OSI metrics a stringent approach was undertaken. For a voxel to be regarded as DS or OS mutually exclusive criteria were employed: DS if DSI > 0.5 and OSI < 0.5; and OS if OSI > 0.5 and

DSI < 0.5. In both cases the goodness of fit for DSI and OSI respectively had to be greater than 0.8, thus the fitted curves explained at least 80% of the integral responses.

Statistical Analyses

The statistical significance of the differences between mean values among animal groups was determined by one-way analysis of variance (ANOVA) followed by Tukey's HSD test, using SigmaPlot (Systat Software). The criterion for statistical significance was set at $p < 0.05$ and all results are represented as mean ± SEM. The statistical significance of the difference in the proportion of diffuse RGCs among groups was determined by chi-squared test, using SigmaPlot (Systat Software). The criterion for statistical significance was set at $p < 0.05$.

SUPPLEMENTAL REFERENCES

Ben Fredj, N., Hammond, S., Otsuna, H., Chien, C.B., Burrone, J., and Meyer, M.P. (2010). Synaptic activity and activity-dependent competition regulates axon arbor maturation, growth arrest, and territory in the retinotectal projection. J. Neurosci. *30*, 10939-10951.

Lister, J.A., Robertson, C.P., Lepage, T., Johnson, S.L., and Raible, D.W. (1999). nacre encodes a zebrafish microphthalmia-related protein that regulates neural-crest-derived pigment cell fate. Development *126*, 3757-3767.

Lowe, A.S., Nikolaou, N., Hunter, P.R., Thompson, I.D., and Meyer, M.P. (2013). A systems-based dissection of retinal inputs to the zebrafish tectum reveals different rules for different functional classes during development. J. Neurosci. *33*, 13946-13956.

Meyer, M.P., and Smith, S.J. (2006). Evidence from in vivo imaging that synaptogenesis guides the growth and branching of axonal arbors by two distinct mechanisms. J. Neurosci. *26*, 3604-3614.

Niell, C.M., and Stryker, M.P. (2008). Highly selective receptive fields in mouse visual cortex. J. Neurosci. *28*, 7520-7536.

Nikolaou, N., Lowe, A.S., Walker, A.S., Abbas, F., Hunter, P.R., Thompson, I.D., and Meyer, M.P. (2012). Parametric functional maps of visual inputs to the tectum. Neuron *76*, 317-324.

Swindale, N.V. (1998). Orientation tuning curves: empirical description and estimation of parameters. Biol. Cybern. *78*, 45-56.

Thisse, C., and Thisse, B. (2008). High-resolution in situ hybridization to whole-mount zebrafish embryos. Nat. Protoc. *3*, 59-69.
