# Supplementary figures and images for: Teneurin-3 Specifies Morphological and Functional Connectivity of Retinal Ganglion Cells in the Vertebrate Visual System
Source: Cell Rep. 2013 Oct 31;5(3):582–92. doi: 10.1016/j.celrep.2013.09.045 (PMC3898612; doi:10.1016/j.celrep.2013.09.045)

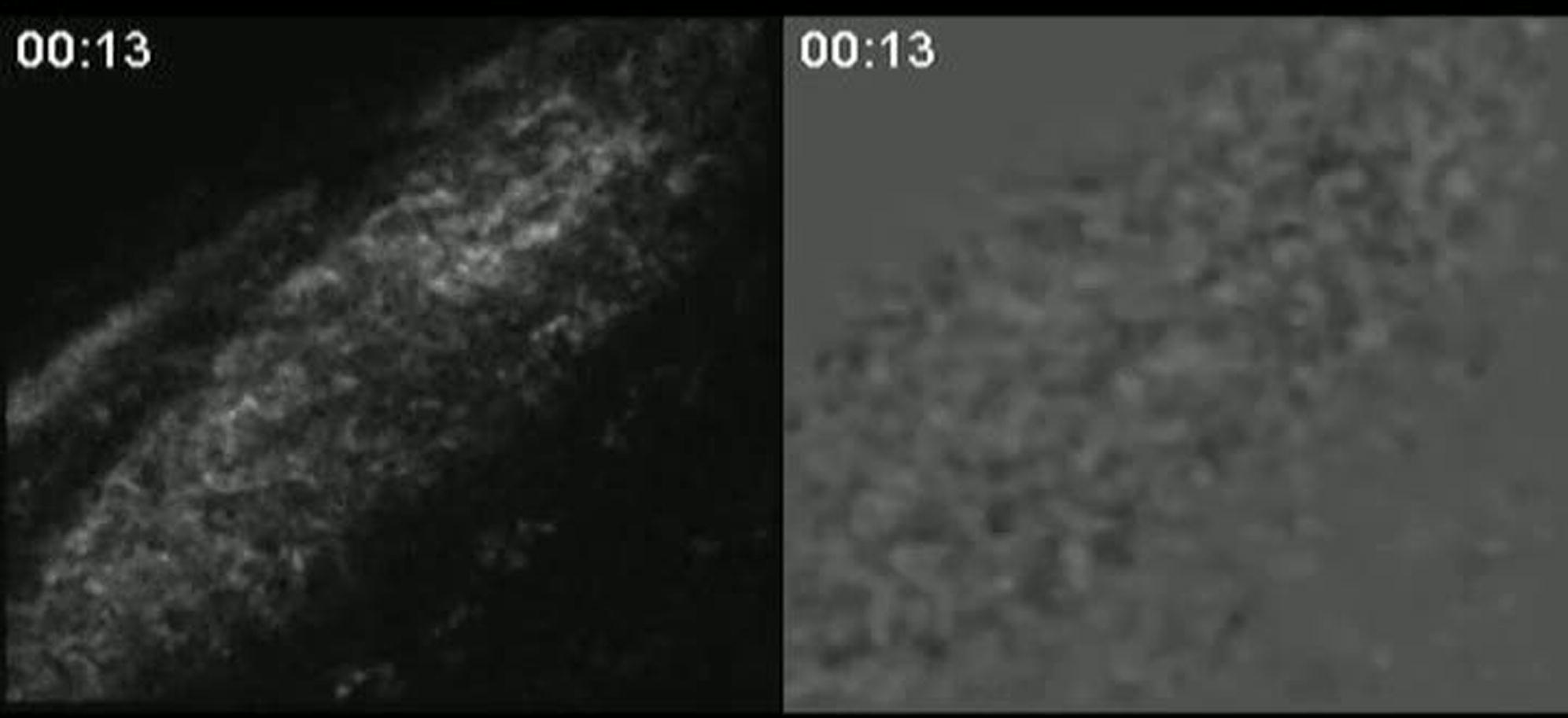

Supplement: Movie S1. Functional RGC Responses to Drifting Bars in a Representative 5 dpf WT Zebrafish Larva, Related to Figures 5 and S2 — Time-lapse functional data of a representative 5 dpf WT larva. The movie encompasses an entire tuning experiment in which all 12 directions of bar motion plus a blank screen null stimulus are presented to one eye of the immobilized zebrafish larva. Unprocessed SyGCaMP3 responses are shown on the left, whereas ΔF/F responses are reported on the right. Time given in min:sec. The same data are presented as parametric map and montage in Figures S2A and S2D, respectively. [file mmc2.jpg]

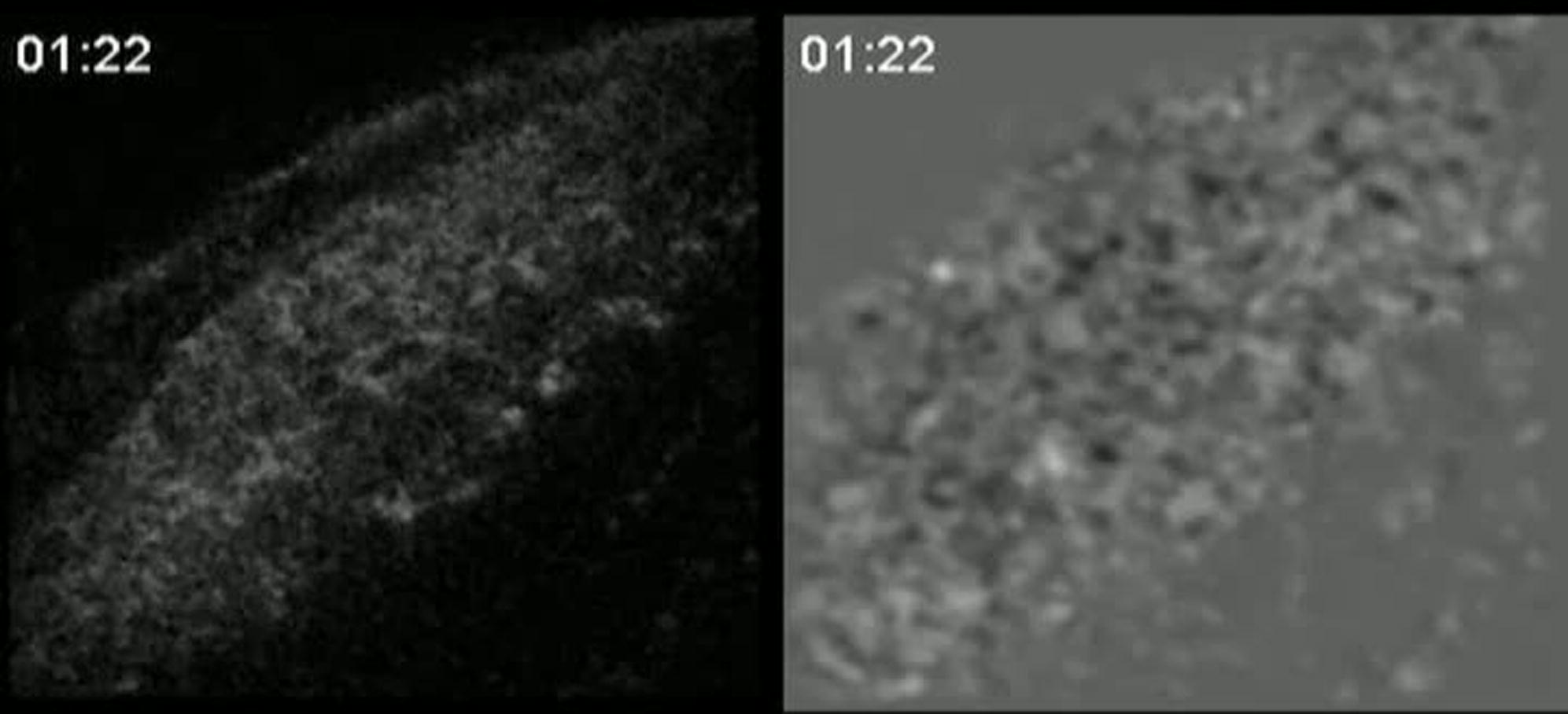

Supplement: Movie S2. Functional RGC Responses to Drifting Bars in a Representative 5 dpf Control MO-Injected Zebrafish Larva, Related to Figures 5 and S2 — Time-lapse functional data of a representative 5 dpf control MO-injected larva. The movie encompasses an entire tuning experiment in which all 12 directions of bar motion plus a blank screen null stimulus are presented to one eye of the immobilized zebrafish larva. Unprocessed SyGCaMP3 responses are shown on the left, whereas ΔF/F responses are reported on the right. Time given in min:sec. The same data are presented as parametric map and montage in Figures S2B and S2E, respectively. [file mmc3.jpg]

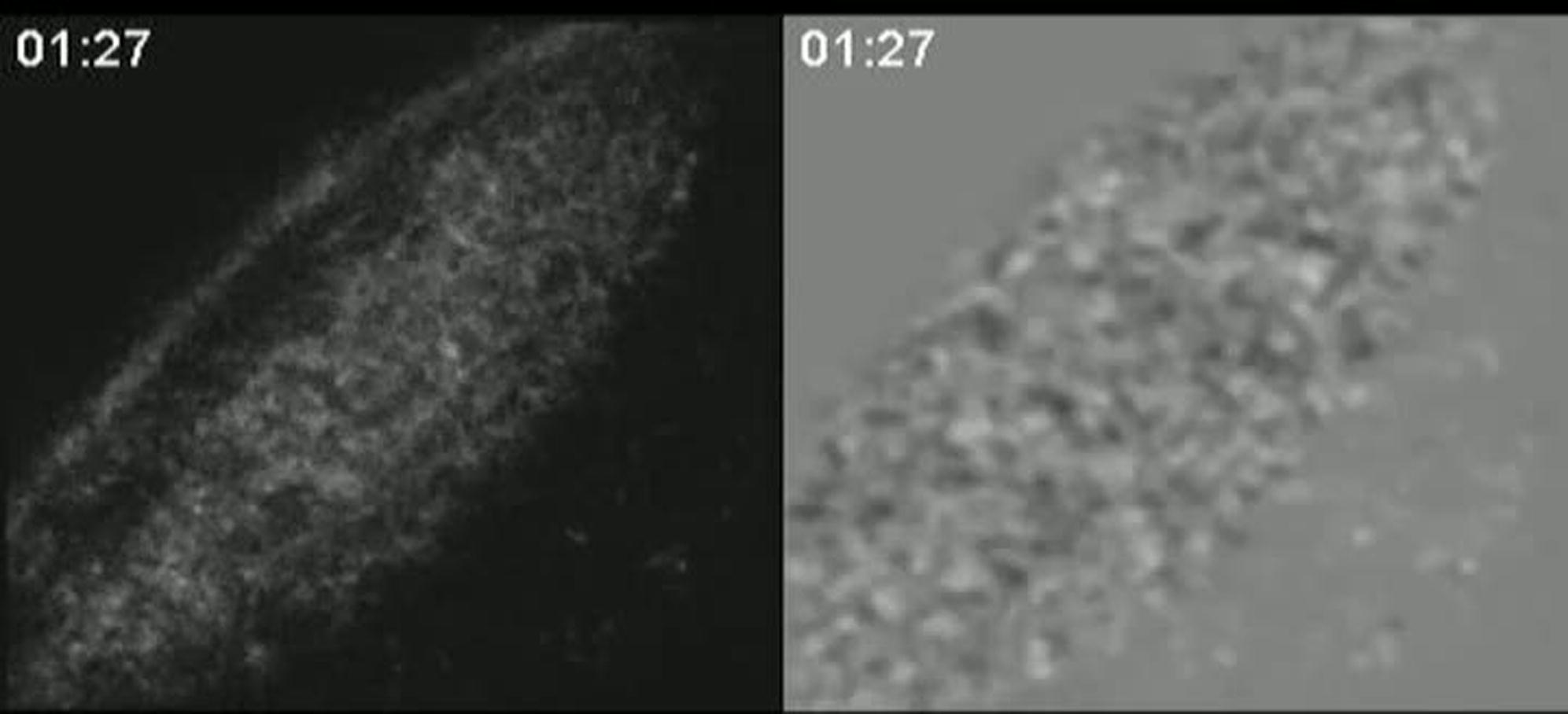

Supplement: Movie S3. Functional RGC Responses to Drifting Bars in a Representative 5 dpf Teneurin-3 Morphant Zebrafish Larva, Related to Figures 5 and S2 — Time-lapse functional data of a representative 5 dpf tenm3 morphant larva. The movie encompasses an entire tuning experiment in which all 12 directions of bar motion plus a blank screen null stimulus are presented to one eye of the immobilized zebrafish larva. Unprocessed SyGCaMP3 responses are shown on the left, whereas ΔF/F responses are reported on the right. Time given in min:sec. The same data are presented as parametric map and montage in Figures S2C and S2F, respectively. [file mmc4.jpg]
