# Supplementary material for: Teneurin-3 Specifies Morphological and Functional Connectivity of Retinal Ganglion Cells in the Vertebrate Visual System
Source: Cell Rep. 2013 Oct 31;5(3):582–92. doi: 10.1016/j.celrep.2013.09.045 (PMC3898612; doi:10.1016/j.celrep.2013.09.045)
Supplement: Document S2. Article plus Supplemental Information [file mmc5.pdf]

Cell Press
Open ACCESS
Cell Reports
Report

Teneurin-3 Specifies Morphological and Functional Connectivity of Retinal Ganglion Cells in the Vertebrate Visual System

Paride Antinucci,[1] Nikolas Nikolaou,[1] Martin P. Meyer,[1] and Robert Hindges[1,*]
[1]MRC Centre for Developmental Neurobiology, King's College London, Guy's Campus, London SE1 1UL, UK
*Correspondence: robert.hindges@kcl.ac.uk
http://dx.doi.org/10.1016/j.celrep.2013.09.045
This is an open-access article distributed under the terms of the Creative Commons Attribution License, which permits unrestricted use, distribution, and reproduction in any medium, provided the original author and source are credited.

SUMMARY

A striking feature of the CNS is the precise wiring of its neuronal connections. During vertebrate visual system development, different subtypes of retinal ganglion cells (RGCs) form specific connections with their corresponding synaptic partners. However, the underlying molecular mechanisms remain to be fully elucidated. Here, we report that the cell-adhesive transmembrane protein Teneurin-3 (Tenm3) is required by zebrafish RGCs for acquisition of their correct morphological and functional connectivity in vivo. Teneurin-3 is expressed by RGCs and their presynaptic amacrine and postsynaptic tectal cell targets. Knockdown of Teneurin-3 leads to RGC dendrite stratification defects within the inner plexiform layer, as well as mistargeting of dendritic processes into outer portions of the retina. Moreover, a subset of RGC axons exhibits tectal laminar arborization errors. Finally, functional analysis of RGCs targeting the tectum reveals a selective deficit in the development of orientation selectivity after Teneurin-3 knockdown. These results suggest that Teneurin-3 plays an instructive role in the functional wiring of the vertebrate visual system.

INTRODUCTION

In the vertebrate retina, retinal ganglion cells (RGCs) develop stereotypic dendritic arborization patterns and make specific synaptic connections with amacrine and bipolar cells in the inner plexiform layer (IPL) (Masland, 2012). The formation of such precise connections is critical for the processing of visual information and the generation of feature selectivity in RGCs (Gollisch and Meister, 2010; Wässle, 2004). A key structural characteristic of visual circuits is the organization of connections into precise laminae (Roska and Werblin, 2001; Sanes and Zipursky, 2010). Recent studies have shown that the assembly of neuropil strata in the IPL is regulated by both adhesive (Yamagata and Sanes, 2008) and repulsive transmembrane proteins (Matsuoka et al., 2011). Similarly, such attractive and repulsive cues are also crucial in establishing specific connectivity between RGC axons and their targets in the brain (Osterhout et al., 2011; Xiao et al., 2011). Our understanding of the molecular mechanisms that specify connections within the retina and between the retina and retinorecipient nuclei in the brain, however, is still far from complete.

Teneurins (Ten-m/Odz) are a phylogenetically conserved family of type II transmembrane proteins (Tucker et al., 2012; Tucker and Chiquet-Ehrismann, 2006). Their large extracellular domain contains eight epidermal growth factor (EGF)-like repeats, multiple tyrosine and aspartate (YD) repeats and five NHL (NCL-1, HT2A, and Lin-41) repeats, which mediate homophilic recognition and adhesion (Beckmann et al., 2013). In vertebrates, these proteins are encoded by four genes, *teneurin 1–4* (also called *odz1–4*), expressed in distinct and often interconnected regions of the nervous system (Tucker and Chiquet-Ehrismann, 2006). In *Drosophila*, the role of teneurins in synaptic partner matching and target choice has been elegantly shown in the olfactory system (Hong et al., 2012) and at the neuromuscular junction (Mosca et al., 2012). In mice, teneurins regulate the generation of binocular visual circuits by controlling the development of ipsilaterally projecting RGCs (Dharmaratne et al., 2012; Leamey et al., 2007; Young et al., 2013). However, a role for teneurins in mediating synapse-specific functional wiring in the vertebrate visual system has yet to be demonstrated.

Here, we investigate the role of *teneurin-3* (hereafter referred to as *tenm3*) in shaping the morphological and functional connectivity of RGCs in vivo using zebrafish. We report that *tenm3* is expressed in RGCs, amacrine cells, and the main retinorecipient target in the brain, the optic tectum. We show that *tenm3* knockdown induces stratification and targeting errors of both dendrites and axons in a subset of RGCs. In support of this, we provide evidence showing that orientation-selective, but not direction-selective, responses are impaired in *tenm3* morphants, suggesting that *tenm3* is involved in wiring subsets of functionally defined visual circuits.

RESULTS

Our study focused on time points between 2 days postfertilization (dpf) and 5 dpf, a period during which RGCs undergo a rapid

Cell PRESS
Open ACCESS

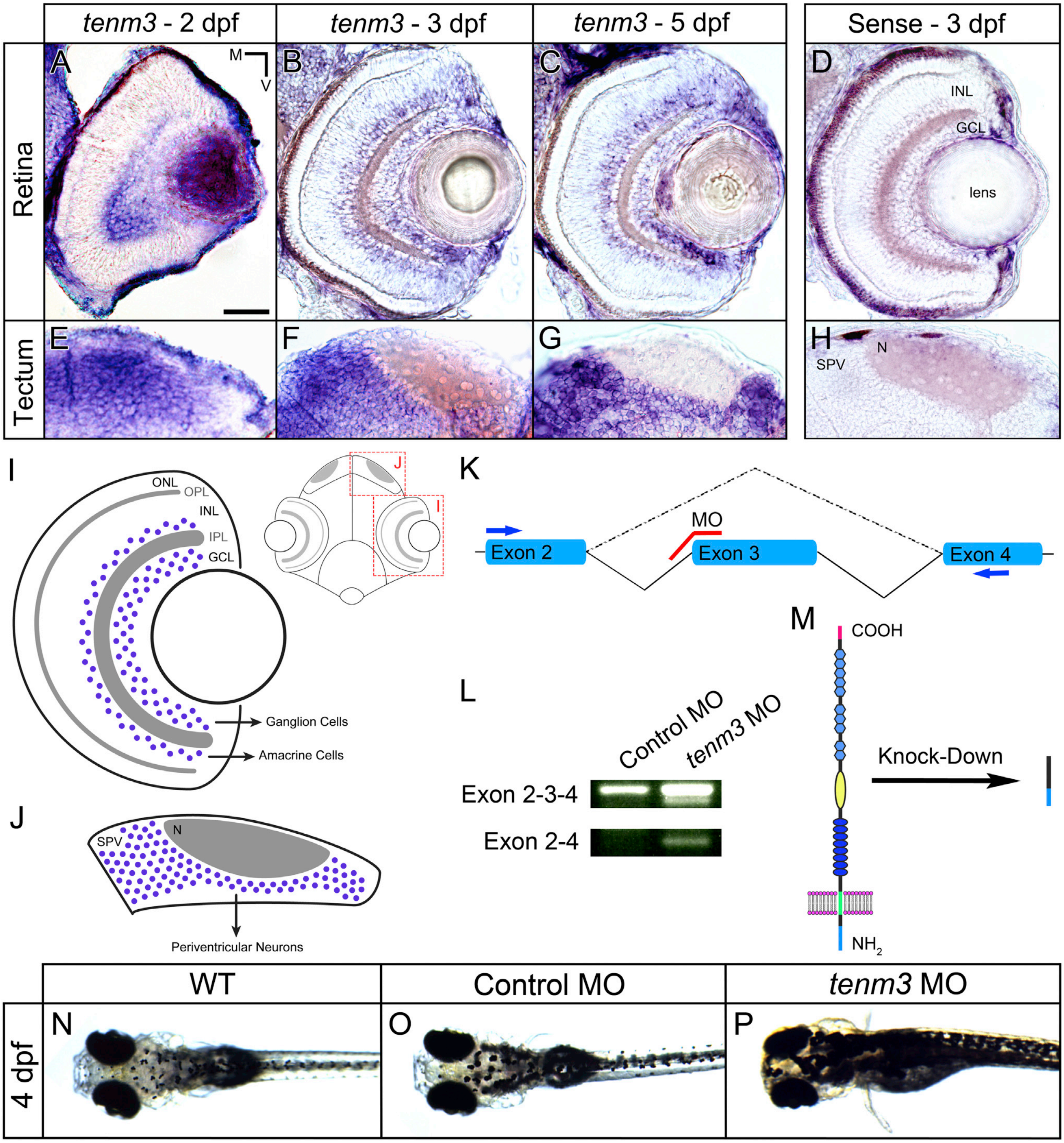

Figure 1. *Teneurin-3* Is Expressed in Interconnected Regions of the Zebrafish Visual System

(A–C) Retinal cryosections of whole-mount in situ hybridizations showing *tenm3* mRNA expression at 2, 3, and 5 dpf.

(D and H) Control in situ hybridizations using sense *tenm3* riboprobe.

(E–G) Tectal cryosections of whole-mount in situ hybridizations showing *tenm3* mRNA expression at 2, 3, and 5 dpf. All images are in transverse plane. Scale bar, 40 μm. N, neuropil; M, medial; V, ventral.

(I) Schematic showing the expression pattern of *tenm3* in the retina. *Tenm3*-positive cells are represented as blue circles. Neuropil layers are indicated in gray. Anatomical reference is reported on the right. IPL, inner plexiform layer; ONL, outer nuclear layer; OPL, outer plexiform layer.

(J) Schematic showing the expression pattern of *tenm3* in the optic tectum.

(K) Schematic detailing the targeting site of splice-blocking *tenm3* morpholino (MO), which is shown in red. Exons are represented in cyan. Solid lines indicate introns. The dashed line indicates exon 3 deletion caused by *tenm3* MO injections. Primers used for RT-PCR (L) are reported as blue arrows.

(legend continued on next page)

phase of morphological and functional development (Lowe et al., 2013; Meyer and Smith, 2006; Mumm et al., 2006).

Teneurin-3 Is Expressed in Interconnected Regions of the Developing Visual System

To reveal the expression pattern of *tenm3* in the developing zebrafish visual system, we carried out in situ hybridization analyses using a specific digoxigenin-labeled antisense riboprobe against *tenm3*. In the retina, *tenm3* is expressed in the ganglion cell layer (GCL) and the inner third of the inner nuclear layer (INL), where amacrine cells are located (Figures 1A–1C). Since in zebrafish only a very small number of displaced amacrine cells reside in the GCL (Connaughton et al., 1999), the majority of signal detected in this layer can be attributed to RGCs. At 2 dpf, *tenm3* is expressed more strongly in the ventral part of the retina (Figure 1A). At 3 and 5 dpf, *tenm3* acquires a sparse expression pattern, suggesting that at these stages of development only a subset of cells are *tenm3*-positive (Figures 1B and 1C). *Tenm3* is also expressed in the main target of RGC axons, the optic tectum (Figures 1E–1G). At 2 dpf, *tenm3* is highly expressed in the medial portion of the stratum periventriculare (SPV), where cell bodies of most tectal cells are located (Figure 1E). Between 3 and 5 dpf, this medial-to-lateral gradient gradually decreases (Figures 1F and 1G) and, at 5 dpf, *tenm3* shows a salt-and-pepper expression pattern (Figure 1G). In summary, *tenm3* is expressed by RGCs, amacrine cells, and tectal neurons (Figures 1I and 1J), consistent with a possible role of *tenm3* in instructing connectivity along the visual pathway.

Teneurin-3 Regulates RGC Dendritic Stratification in the IPL

To investigate the function of *tenm3* within the developing visual system, we used antisense morpholino oligonucleotides (MOs) to knock down *tenm3* expression levels. We designed a splice-blocking MO (Draper et al., 2001) targeting the boundary between intron 2 and exon 3 (hereafter referred to as *tenm3* MO; Figure 1K). Injection of *tenm3* MO into one-cell-stage zebrafish embryos produces the deletion of exon 3 (Figure 1L), which encodes part of the intracellular domain. This leads to a frameshift in exon 4 (transmembrane domain) and a subsequent early stop codon in exon 5, resulting in deletion of the transmembrane and extracellular domains (Figure 1M). To confirm results obtained with this *tenm3* MO, a second splice-blocking MO targeting a nonoverlapping region of *tenm3* mRNA (i.e., the boundary between exon 4 and intron 4) was also used (*tenm3* MO 2; see Figure S1). *Tenm3* morphants are viable and do not show any obvious morphological defect. However, 4 dpf *tenm3* MO-injected larvae fail to show a normal visually mediated background adaptation (VBA) and therefore appear darker compared to wild-type (WT) and control MO-injected larvae (Figures 1N–1P). Since the VBA is a neuroendocrine response dependent on the function of RGCs (Kay et al., 2001), we deduced that the knockdown of *tenm3* somehow impairs the normal development of the visual system.

In order to examine IPL organization in vivo, we used the Tg(*Isl2b:Gal4;UAS:Kaede*) transgenic zebrafish line (see Experimental Procedures), where the fluorescent protein Kaede is expressed in the majority of RGCs. At 5 dpf, when RGC dendrites exhibit clear stratification, four Kaede-positive strata are visible in the IPL of WT and control MO-injected larvae (Figures 2A and 2B). Fluorescence intensity measurements across the IPL of multiple larvae show that these strata are positioned at 5%, 33%, 66%, and 95% depth of the IPL (with 0% corresponding to GCL/IPL border and 100% to IPL/INL border), and thus were named S5, S33, S66, and S95, respectively (Figure 2E; WT n = 7 larvae, control MO n = 7). The presence of four dendritic strata in the IPL of 5 dpf zebrafish larvae is consistent with previous work using the Tg(*Brn3c:MGFP*) transgenic line, where approximately 50% of RGCs are labeled (Mumm et al., 2006). In 5 dpf *tenm3* morphants, by contrast, strata within the IPL are poorly defined (Figures 2C and 2D). The average fluorescence intensity profile reveals that only three Kaede-positive strata are present in the IPL of *tenm3* morphants (Figure 2E; n = 10 larvae). Specifically, only one irregularly laminated stratum is visible in the medial portion of the IPL, instead of the two middle strata (S33 and S66) found in WT and control MO retinae. Furthermore, the outermost stratum (S95) is not tightly stratified and appears thicker compared to control groups. No significant difference in IPL width was observed among the three groups (WT 15.2 ± 0.2 μm; control MO 15.0 ± 0.1 μm; *tenm3* MO 15.1 ± 0.2 μm; $F_{2,21} = 0.08$, $p = 0.92$, n = 24 larvae). In addition to these stratification abnormalities in the IPL, we detected ectopic RGC processes in the INL of *tenm3* morphants (Figures 2C′ and 2D′, cyan arrowheads; n = 19 out of 20 larvae), a phenomenon never observed in WT and control MO larvae, where all RGC dendrites are confined within the IPL (Figures 2A′ and 2B′; n = 10 larvae per group). Strikingly, in some cases, these processes reach the outer plexiform layer (OPL; Figure 2C′, yellow arrow). Ectopic RGC processes extending into the INL were also seen in *tenm3* morphant retinae at 3 dpf, when RGCs start to develop stratified dendritic arbors within the IPL (data not shown).

To resolve the changes in RGC dendritic morphology in greater detail, we mosaically labeled individual RGCs by coinjecting *Ath5:Gal4*, *UAS:GFP* and *UAS:tdTomato* DNA constructs into one-cell-stage embryos. The combinatorial expression of different fluorescent reporters in RGCs enabled us to distinguish between occasionally overlapping dendritic arbors of different cells. Using this approach, we were able to determine that the neurites mistargeting into outer layers of the retina observed in *tenm3* morphants originate from RGC dendrites (Figure 3A, cyan arrowheads) and that this phenotype is restricted to a

(L) RT-PCR analysis of *tenm3* mRNA structure in control MO- and *tenm3* MO-injected embryos. Two shorter splice variants are distinguished in *tenm3* morphants. cDNA sequence comparison revealed that the shortest splice variant lacks exon 3.
(M) Schematic detailing the effect of exon 3 deletion caused by the splice-blocking *tenm3* MO, resulting in the deletion of Tenm3 transmembrane and extracellular domains. The full-length protein is represented on the left. The N terminus is located intracellularly, whereas the C terminus is in the extracellular space.
(N–P) At 4 dpf, *tenm3* morphant larvae fail to visually adapt their skin pigmentation to the level of background illumination.
See also Figure S1.

Cell PRESS
Open ACCESS

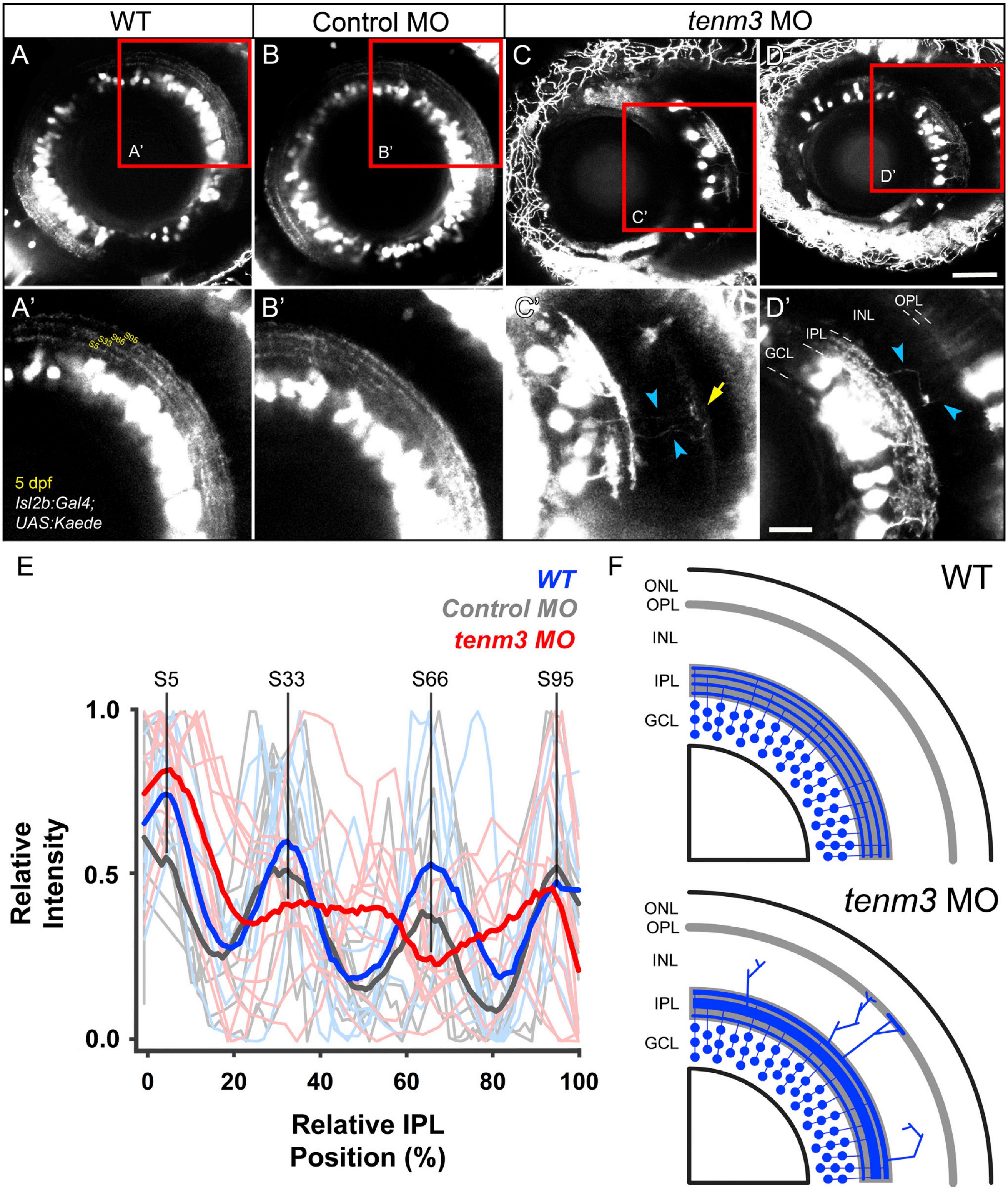

Figure 2. *Teneurin-3* Is Required for Correct Stratification of RGC Dendrites

(A–D) Kaede-expressing RGCs in the retina of 5 dpf WT, control MO-injected, and *tenm3* MO-injected larvae.

(A′–D′) Insets in (A)–(D) showing the dendritic stratification pattern of Kaede-positive RGCs. All images represent maximum intensity projections of ~20 μm confocal z stacks. Scale bars, 40 μm (A–D) and 20 μm in (A′–D′). GCL, ganglion cell layer; INL, inner nuclear layer; IPL, inner plexiform layer; OPL, outer plexiform layer.

(legend continued on next page)

Cell PRESS
Open ACCESS

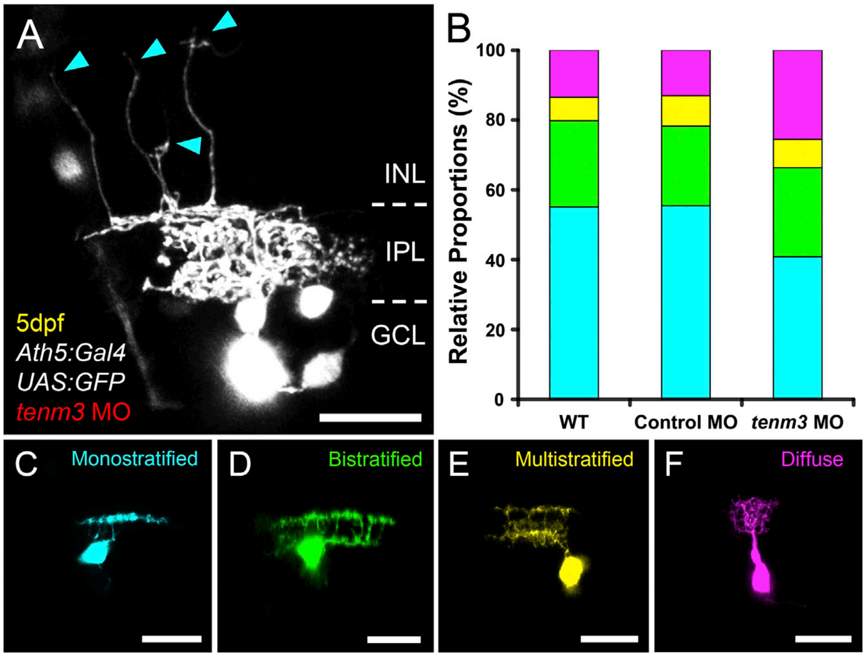

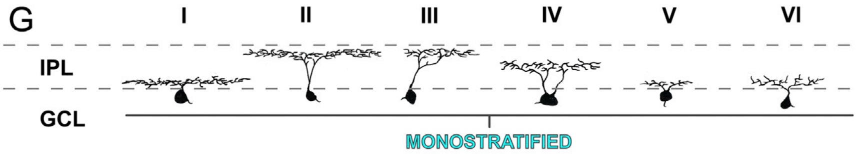

	I	II	III	IV	V	VI
WT	7.9% (7)	3.4% (3)	6.7% (6)	11.2% (10)	10.1% (9)	15.7% (14)
Control MO	6.5% (6)	3.3% (3)	8.7% (8)	11.9% (11)	10.9% (10)	14.1% (13)
tenm3 MO	8.2% (8)	0% (0)	8.2% (8)	5.1% (5)	7.1% (7)	12.2% (12)

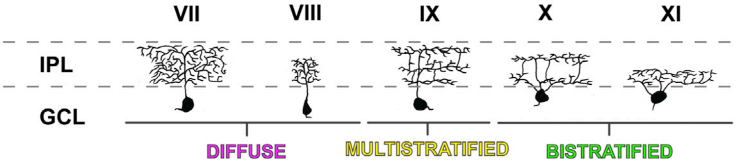

	VII	VIII	IX	X	XI
WT	5.6% (5)	7.9% (7)	6.7% (6)	21.3% (19)	3.4% (3)
Control MO	4.3% (4)	8.7% (8)	8.7% (8)	19.6% (18)	3.3% (3)
tenm3 MO	10.2% (10)	11.2% (11)	8.2% (8)	22.4% (22)	3.1% (3)

Figure 3. Higher Proportion of RGCs with Diffuse Dendritic Arbors in *teneurin-3* Morphants

(A) Lateral view of mosaically labeled RGCs in the retina of a 5 dpf *tenm3* MO-injected larva. Scale bar, 20 μm. GCL, ganglion cell layer; INL, inner nuclear layer; IPL, inner plexiform layer.

(B) Bar graph showing the proportions of 5 dpf RGCs possessing monostratified (cyan, C), bistratified (green, D), multistratified (yellow, E), and diffuse (magenta, F) dendritic arbors relative to the total number mosaically labeled RGCs within each animal group (WT n = 89 cells in 34 larvae; control MO n = 92 cells in 39 larvae; *tenm3* MO n = 98 cells in 49 larvae).

(C–F) Representative RGCs with monostratified (C), bistratified (D), multistratified (E), and diffuse (F) dendritic arbors. All images represent maximum intensity projections of ~30 μm confocal z stacks that have been pseudocolored and rotated to best show dendritic arborizations. Scale bars, 20 μm.

(G) Summary table showing the morphological classification and frequency of the 11 RGC types within each group (number of cells found per each type are reported in brackets). In *tenm3* morphants, four diffuse RGCs (4.1% of cells) showed dendritic arborization patterns that could not be classified in any of the 11 types and, hence, were not included in the table.

subset of cells (n = 5 cells out of 98 in 49 larvae). Moreover, mosaic labeling allowed us to visualize the precise IPL dendritic stratification patterns of single RGCs (Figures 3C–3F). Interestingly, 5 dpf *tenm3* morphants show a significantly higher proportion of RGCs possessing diffuse dendritic arbors (*tenm3* MO 25 diffuse versus 73 stratified cells in 49 larvae; WT 12 diffuse versus 77 stratified cells in 34 larvae; control MO 12 diffuse versus 80 stratified cells in 39 larvae; $\chi^2 = 6.596$, df = 2, p = 0.037). Looking at the relative proportions between monostratified, bistratified, multistratified, and diffuse RGCs, it appears that the increase in number of RGCs with diffuse dendritic arbors is exclusively at the expense of monostratified RGCs (Figure 3B; WT 55.1% monostratified, 24.7% bistratified, 6.7% multistratified, 13.5% diffuse; control MO 55.5% monostratified, 22.8% bistratified, 8.7% multistratified, 13% diffuse; *tenm3* MO 40.8% monostratified, 25.5% bistratified, 8.2% multistratified, 25.5% diffuse). Further identification and classification of the 11 RGC types previously reported in the adult zebrafish retina (Mangrum et al., 2002) revealed that the monostratified RGC types are not indiscriminately affected by *tenm3* knockdown. In fact, some RGC monostratified types decrease in frequency in *tenm3* morphants whereas others show frequencies comparable to those found in control animals (Figure 3G). Overall, these data show that *tenm3* knockdown causes structural irregularities in the developing

(E) Fluorescence profiles of IPL stratification in 5 dpf WT (blue), control MO-injected (gray), and *tenm3* MO-injected (red) larvae. Thin traces represent intensity profiles of IPLs of single larvae. Thick traces indicate average profiles (WT, n = 7 larvae; control MO, n = 7; *tenm3* MO, n = 10). Zero percent corresponds to the boundary between GCL and IPL, whereas 100% corresponds to the boundary between IPL and INL.

(F) Schematic summarizing the defects observed in *tenm3* morphant retinae. RGCs are indicated in blue. Neuropil layers are in gray. ONL, outer nuclear layer. See also Figure S4.

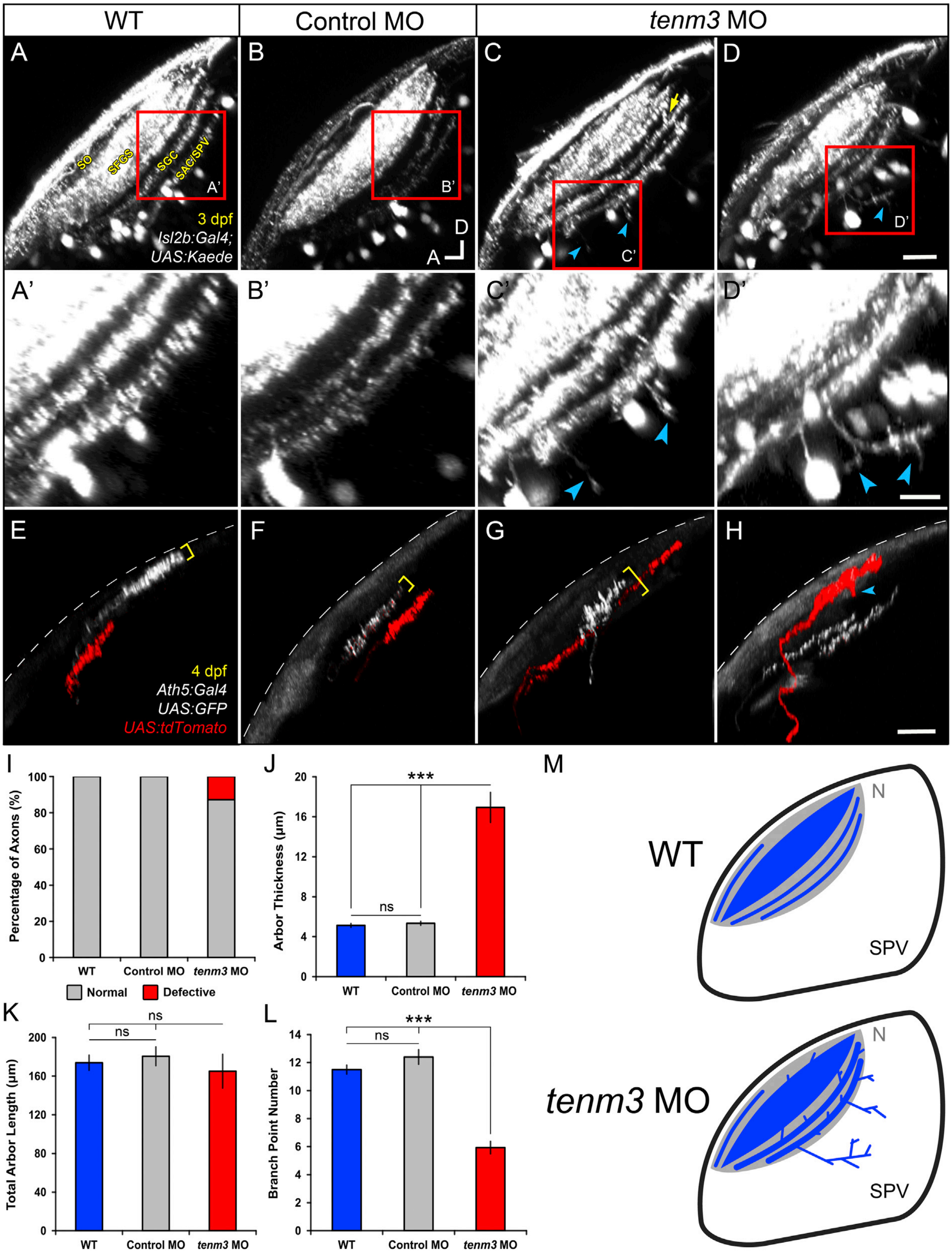

(legend on next page)

retina (Figure 2F) and that changes in RGC dendritic stratification appear to be limited to specific RGC types.

Laminar Targeting Errors in a Subset of RGC Axons Upon Teneurin-3 Knockdown

We next examined RGC axonal arborization in the tectal neuropil. Similar to the IPL in the retina, this structure is characterized by a stereotypic lamination pattern (Xiao et al., 2011). Using the Tg(*Isl2b:Gal4;UAS:Kaede*) zebrafish line, we visualized the four main retinorecipient laminae of the tectum that, from the most superficial to the deepest, are named stratum opticum (SO), stratum fibrosum et griseum superficiale (SFGS), stratum griseum centrale (SGC), and lamina at the interface between the stratum album centrale and the stratum periventriculare (SAC/SPV; Figure 4A) (Nevin et al., 2010). In 3 dpf WT and control MO larvae, all RGC axons are restricted to these four laminae and no axons are found outside the neuropil region (Figures 4A and 4B; n = 15 larvae per group). In *tenm3* morphants, by contrast, we observed neurites projecting aberrantly into the SPV (Figures 4C and 4D, cyan arrowheads; n = 18 out of 23 larvae). 3D reconstruction and neurite tracing revealed that these processes arise principally from the deepest lamina (SAC/SPV) and, in some cases, are up to 30–40 μm long and possess several branches (Figure 4D′, cyan arrowheads). In addition, tectal laminae of *tenm3* morphants are less precisely delimited and axons aberrantly cross lamina borders (Figure 4C, yellow arrow).

To examine in more detail how the lamination defects seen at the population level arise, we labeled individual RGCs through mosaic expression of either GFP or tdTomato driven by the *ath5* promoter. As a rule, individual RGC axons arborize in a planar fashion within a single tectal lamina or sublamina (the SO and SFGS are further subdivided into 2 and 6 sublaminae, respectively) (Robles et al., 2013). This behavior was confirmed in 4 dpf control groups, where 100% of labeled axons (WT n = 102 axons in 50 larvae; control MO n = 94 axons in 45 larvae) showed planar arborization patterns (Figures 4E, 4F, 4I, and 4J; arbor thickness WT 5.1 ± 0.1 μm; control MO 5.3 ± 0.1 μm; n = 20 axons per group). In contrast, we found RGCs with abnormally laminated axonal arbors in *tenm3* morphants (Figures 4G and 4H). Intriguingly, these axons represent only a fraction of the total number of labeled RGCs (Figure 4I; 12.7%, n = 20 axons out of 157 in 80 larvae). They are characterized by possessing axonal processes projecting toward adjacent laminae (Figure 4H, cyan arrowhead) and significantly broader cross-sectional profiles (arbor thickness *tenm3* MO 16.9 ± 1.4 μm; $F_{2,57}$ = 57.97, $p < 0.0001$, n = 20 axons) than those observed in control animals (Figures 4G and 4J). The total arbor length of aberrant axons is comparable to that of control groups (Figure 4K; *tenm3* MO 165.1 ± 17.4 μm; WT 173.8 ± 7.7 μm; control MO 180.5 ± 9.7 μm; $F_{2,57}$ = 0.33, p = 0.72, n = 20 axons per group) but their number of branch points is significantly lower (Figure 4L; *tenm3* MO 5.9 ± 0.4; WT 11.5 ± 0.3; control MO 12.4 ± 0.5; $F_{2,57}$ = 48.86, $p < 0.0001$, n = 60), suggesting that *tenm3* knockdown impairs their capacity to either form or stabilize new branches, without affecting overall arbor length. Taken together, these results indicate that *tenm3* is required for the correct laminar targeting and arborization of a subset of RGC axons (Figure 4M).

Teneurin-3 Is Required for Functional Development of Orientation-Selective RGCs

To investigate the functional consequences of *tenm3* knockdown, we analyzed direction-selective (DS) and orientation-selective (OS) responses of RGC axon terminals innervating the tectal neuropil. Light or dark drifting bars moving in 12 directions were presented to one eye of 5 dpf Tg(*Isl2b:Gal4;UAS:SyGCaMP3*) transgenic larvae while functionally imaging the contralateral tectum (Figure 5A) (Nikolaou et al., 2012). Since SyGCaMP3 is based on the fusion between the synaptic vesicle protein synaptophysin and the genetically encoded calcium indicator GCaMP3, this transgenic line enables the targeting of the probe specifically to RGC presynaptic terminals and hence the functional analysis of RGCs within the tectal target. RGCs of all three animal groups respond to drifting bars (Movies S1, S2, and S3) and exhibit complex patterns of stimulus responses (Figure S2). In order to characterize and map visual response properties (i.e., direction and orientation selectivity) present in the retinal input to the tectum, we used a voxel-wise analysis strategy that is independent of cellular and neuropil morphology (Nikolaou et al., 2012). Only visually responsive voxels were subjected to further characterization. Direction- and orientation-selective indices (DSI and OSI) based on fitted von Mises profiles were calculated together with an estimate for their goodness of fit, R^2 (Lowe et al., 2013) (see Supplemental Experimental Procedures). For a voxel to be regarded as DS or OS, mutually exclusive criteria were employed: DS if $R^2 > 0.8$, DSI > 0.5, and OSI < 0.5; and OS if $R^2 > 0.8$, OSI > 0.5, and DSI < 0.5 (Figure 5B). Functional maps in which DS and OS voxels are color-coded, obtained from individual larvae, were spatially coregistered to generate parametric composite maps (Figures 5C–5E; WT n = 8 larvae; control MO n = 11; *tenm3* MO n = 20). Analyzing the

Figure 4. Axon Laminar Targeting Errors in a Subset of RGCs in *teneurin-3* Morphants

(A–D) The four main retinorecipient laminae of the tectum are visible in the Tg(*Isl2b:Gal4;UAS:Kaede*) zebrafish line at 3 dpf. SO, stratum opticum; SFGS, stratum fibrosum et griseum superficiale; SGC, stratum griseum centrale; SAC, stratum album centrale; SPV, stratum periventriculare.

(A′–D′) Insets in (A)–(D) showing RGC axon lamination in deep laminae of the tectal neuropil.

(E–H) Lateral view of mosaically labeled RGC axons at 4 dpf. Dashed lines indicate the skin overlaying the tectum. All images represent maximum intensity projections of ~50 μm confocal z stacks that have been rotated around the longitudinal axis to best show axonal lamination. Scale bars, 20 μm (A–H) and 10 μm in (A′–D′). A, anterior; D, dorsal.

(I) Quantification of axon laminar targeting behaviors in mosaically labeled RGCs (WT n = 102 axons in 50 larvae; control MO n = 94 axons in 45 larvae; *tenm3* MO n = 157 axons in 80 larvae).

(J–L) Bar graphs showing the measurements for arbor thickness (J), total arbor length (K), and branching point number (L) of single RGC axons (n = 20 axons per group). All graphs show mean values ± SEM. ***$p < 0.001$; ns, not significant by one-way ANOVA followed by Tukey's HSD test.

(M) Schematic summarizing the defects observed in the optic tecta of *tenm3* morphants. RGC axons are indicated in blue. Neuropil layers are in gray. N, neuropil.

See also Figure S4.

Cell PRESS Open ACCESS

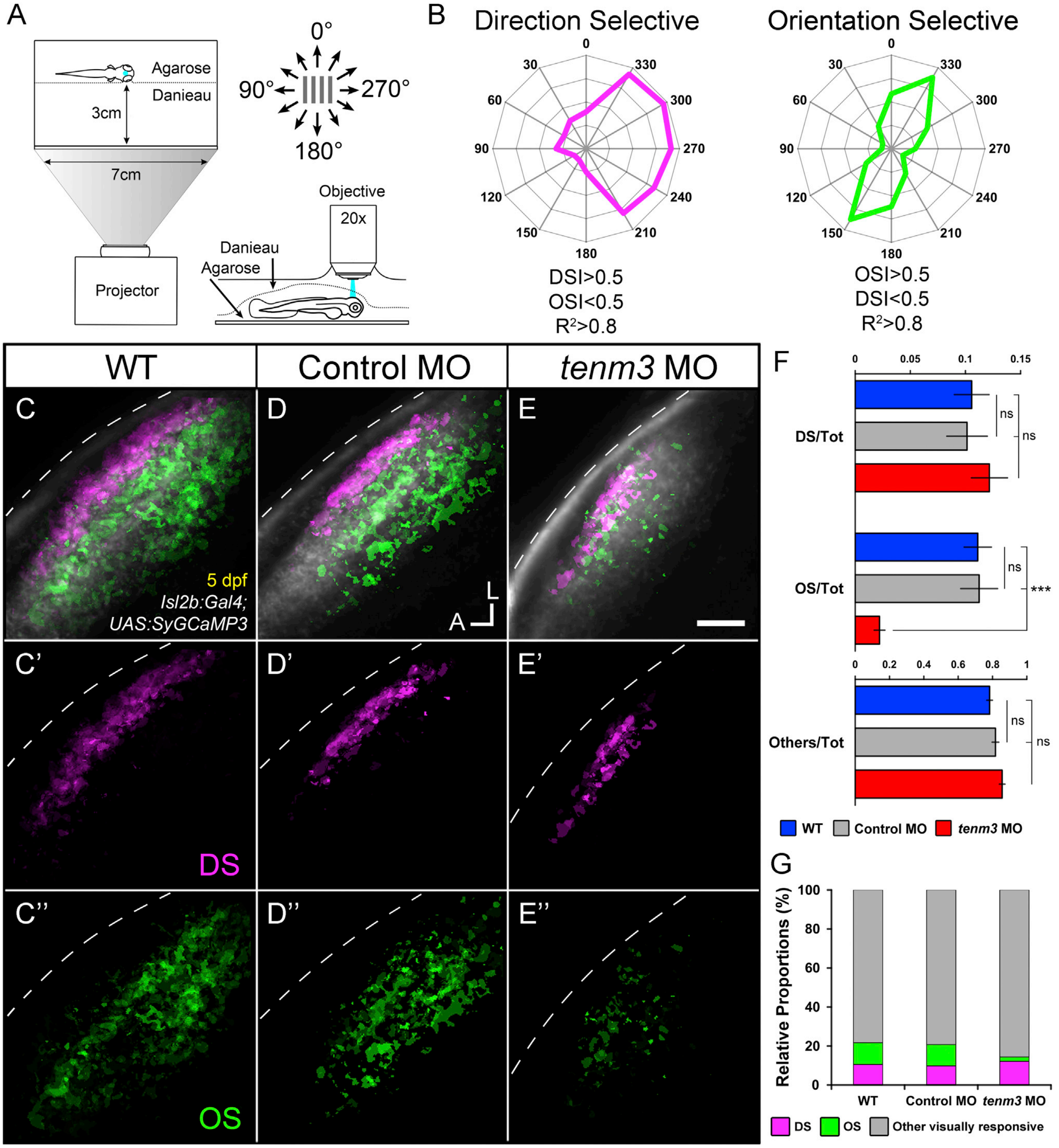

Figure 5. Impaired Development of Orientation-Selective RGCs Following *teneurin-3* Knockdown

(A) Schematic describing the experimental setup. Larvae were immobilized in agarose and placed with one eye facing a screen, where drifting bars moving in 12 directions were projected. Visually evoked SyGCaMP3 responses were recorded in the contralateral tectal neuropil.

(B) Polar plots of representative direction-selective (DS, magenta) and orientation-selective (OS, green) voxels showing relative integral responses to moving bars. Criteria employed to characterize the two classes of voxels are reported at the bottom.

(C–E) Composite parametric maps across multiple 5 dpf Tg(*Isl2b:Gal4;UAS:SyGCaMP3*) larvae representing the spatial distribution of DS (magenta) and OS (green) voxels within each group (WT n = 8 larvae; control MO n = 11; *tenm3* MO n = 20). Within individual parametric maps, voxel brightness is proportional to the summed incidence of each functional response across all larvae imaged. The standard space template image derived for each group (grayscale) provides an anatomical reference. Dashed lines indicate the skin overlaying the tectum. Scale bar, 20 μm. A, anterior; L, lateral.

(legend continued on next page)

DS RGC input to the tectum, we observed that in all three experimental groups DS responses are present (Figures 5C′–5E′). Moreover, the normal laminar organization of DS voxels within the superficial region of SFGS (Nikolaou et al., 2012) is preserved in *tenm3* morphants (Figures 5C′–5E′). Further analysis of DS RGC subtypes revealed that all three DS RGC populations—tuned to anterior (~260°), dorsoposterior (~40°), and ventroposterior (~150°) motion—found in control groups (Nikolaou et al., 2012) are also present in *tenm3* morphants (Figure S3). Overall, no difference between *tenm3* morphants and control groups was observed in the DS RGC input to the tectum.

In contrast, we found that the OS RGC input to the tectum is severely impaired upon *tenm3* knockdown. Specifically, the overall number of OS voxels is decreased in *tenm3* morphants (Figures 5C″–5E″). In addition, the OS RGC voxels that are typically found in deeper sublaminae of SFGS with little or no overlap with DS RGCs in control animals (Figures 5C and 5D) (Nikolaou et al., 2012) show a substantial degree of overlap with DS voxels in *tenm3* morphants (Figure 5E). To further confirm the OS RGC impairment, we analyzed the relative proportions of functional response classes within each experimental group. In *tenm3* morphants, we found a significant decrease in the ratio between OS voxels and the total population of visually responsive voxels (Figure 5F; OS/tot *tenm3* MO 0.022 ± 0.004, n = 20 larvae; WT 0.111 ± 0.012, n = 8; control MO 0.112 ± 0.016, n = 11; $F_{2,36}$ = 24.61, $p < 0.0001$), so the OS input becomes the smallest population of RGCs responding to drifting bars in this group (Figure 5G). The relative proportions of DS and non-DS/non-OS (classified as "others") voxel populations, however, were similar among the three animal groups (Figures 5F and 5G; DS/tot WT 0.105 ± 0.015, control MO 0.101 ± 0.018, *tenm3* MO 0.121 ± 0.016, $F_{2,36}$ = 0.42, p = 0.66; others/tot WT 0.783 ± 0.016, control MO 0.817 ± 0.019, *tenm3* MO 0.856 ± 0.016, $F_{2,36}$ = 3.06, p = 0.059, n = 39 larvae), suggesting no impairment by *tenm3* knockdown. These functional results indicate that visual responses of OS RGCs are affected by *tenm3* knockdown whereas DS RGCs develop normally, therefore reinforcing the possible role of *tenm3* in the assembly of specific visual circuits. All structural and functional phenotypes observed using *tenm3* MO were confirmed in larvae injected with a second splice-blocking MO against *tenm3* (*tenm3* MO 2; Figure S4), supporting the specificity of gene knockdown.

DISCUSSION

Recent studies in *Drosophila* showed that teneurins are involved in establishing specific synaptic circuits (Hong et al., 2012; Mosca et al., 2012). However, a similar role in vertebrate neural circuit wiring has not yet been demonstrated. Here, we report that Teneurin-3 is required for the correct structural and functional development of RGCs in zebrafish. RGCs and their pre- and postsynaptic cellular targets (i.e., amacrine cells and tectal neurons, respectively) express *tenm3* during the period of intense synapse formation (2–5 dpf), suggesting an instructive role in synaptic matching through homophilic interactions between neuronal partners along the visual pathway. *Tenm3* knockdown produces laminar targeting errors of RGC dendrites and axons, indicating that Tenm3 acts in both the IPL of the retina and the tectal neuropil. Intriguingly, these errors appear to be restricted to a subset of RGCs, hinting that Tenm3 acts in specific RGC subtypes and that Tenm3-negative cells are unaffected. Consistent with this hypothesis, when we examined the functional development of visual response properties conveyed by RGCs, we observed that the OS retinal input to the tectum is strongly impaired whereas direction selectivity is not affected in *tenm3* morphant larvae. This does not exclude, however, that additional RGC functional subtypes may be affected in *tenm3* morphants. Previous studies in mice showed that Teneurin-3 regulates the development of topography in the retinogeniculate (Leamey et al., 2007) and retinocollicular pathways (Dharmaratne et al., 2012), specifically for the ipsilaterally projecting RGC population. However, the fact that *teneurin-3* is not exclusively expressed in ipsilaterally projecting RGCs (Leamey et al., 2007) and is also found in the visual system of species where RGCs project contralaterally only, like chick (Kenzelmann-Broz et al., 2010) and zebrafish (Mieda et al., 1999; this study), clearly suggests additional functions in vertebrate visual system development.

Taken together, our findings support a role for Tenm3 in the establishment of functional cell subtype-specific wiring in vertebrates. What developmental mechanisms does Tenm3 regulate? It is generally accepted that molecules mediating homophilic cell-cell adhesion instruct the recognition between pre- and postsynaptic elements by triggering specific synapse formation/stabilization (Sanes and Yamagata, 2009; Williams et al., 2010). In addition, teneurin-mediated homophilic recognition and subsequent formation of cell-adhesion partners leads to inhibition of neurite outgrowth (Beckmann et al., 2013). Thus, the simplest hypothesis is that *tenm3* (by being expressed in RGCs, amacrine cells, and tectal neurons) controls the lamination of RGC neurites through stabilization of branches contacting neurites of *tenm3*-expressing cells. Homophilic adhesion has been extensively studied in the IPL of the chick retina, where different immunoglobulin superfamily adhesion molecules are expressed by specific subsets of cells and control the precise sublaminar matching of their neurites (Yamagata and Sanes, 2008, 2012). Interestingly, this matching mechanism appears to be conserved in higher visual targets. For example, evidence in mouse showed that Cadherin-6 mediates the axon-target recognition between a specific subset of RGCs and their target

(C′–E′) Parametric maps for DS voxels only.
(C″–E″) Parametric maps for OS voxels only.
(F) Bar graphs showing the ratios between defined voxel classes and total visually responsive voxels (Tot) within each group (WT n = 8 larvae; control MO n = 11; *tenm3* MO n = 20). Non-DS and non-OS voxels are classified as "others." All graphs show mean values ± SEM. ***p < 0.001; ns, not significant by one-way ANOVA followed by Tukey's HSD test.
(G) Bar graph showing the proportions of DS and OS voxel classes relative to visually responsive voxels within each group.
See also Figures S2–S4 and Movies S1, S2, and S3.

nuclei in the brain (Osterhout et al., 2011). An alternative mechanism that might regulate RGC neurite arborization is the neurite costratification between morphologically and functionally related cells expressing the same combination of adhesive proteins. This kind of interaction certainly occurs during IPL development. In studies where single or multiple retinal cell classes were selectively eliminated, the remaining cellular components could form a stratified IPL, therefore suggesting that no single pre- or postsynaptic retinal cell class is strictly essential for IPL formation (Kay et al., 2004; Randlett et al., 2013). Further experiments are needed to determine the exact mechanisms of action of Tenm3 and in which cell subtypes it is expressed. Meanwhile, our results presented here point toward an important role for teneurins in the development of vertebrate neural circuit specificity.

EXPERIMENTAL PROCEDURES

Transgenic Lines and Constructs

Transgenic lines Tg(*Isl2b:Gal4*) and Tg(*UAS:SyGCaMP3*) have been described previously (Ben Fredj et al., 2010; Nikolaou et al., 2012). Transgenic line Tg(*UAS:Kaede*) was a gift of Prof. Chi Bin-Chien. The *UAS:GFP* and *UAS:tdTomato* DNA constructs were described previously (Ben Fredj et al., 2010), and the *Ath5:Gal4* plasmid was a gift of Prof. Steve Wilson (UCL, UK). All animal procedures were approved by the local Animal Welfare and Ethics Review Body (King's College London) and were carried out in accordance with the Animals (Scientific Procedures) Act 1986, under license from the United Kingdom Home Office.

Functional Imaging

Confocal imaging was performed using an LSM 710 confocal microscope equipped with a spectral detection scan head and a 20×/1.0 NA water-immersion objective (Carl Zeiss). Functional time series of visually evoked SyGCaMP3 responses were acquired at a rate of 4.1 Hz and 0.415 × 0.415 μm resolution (256 × 256 pixels) and 1 AU pinhole aperture. Visual stimulation and voxel-wise analysis of functional data were performed as described previously (Nikolaou et al., 2012) (see Supplemental Experimental Procedures).

Statistical Analyses

The statistical significance of the differences between mean values and in the proportion of diffuse RGCs among groups was determined by one-way ANOVA followed by Tukey's HSD test and chi-square test, respectively, using SigmaPlot (Systat Software). The criterion for statistical significance was set at $p < 0.05$ and results are represented as mean ± SEM.

SUPPLEMENTAL INFORMATION

Supplemental Information includes Supplemental Experimental Procedures, four figures, and three movies and can be found with this article online at http://dx.doi.org/10.1016/j.celrep.2013.09.045.

ACKNOWLEDGMENTS

We thank L. Ward and J. Clarke for control morpholino, S. Wilson for *Ath5:Gal4* DNA construct, and A. Lowe for software design and instruction. We also thank N. Maiorano for comments on the manuscript and P. Hunter for practical help. This study was supported by a KCL investment grant to R.H. and a KCL Health Schools PhD studentship sponsored by the Medical Research Council (MRC) to P.A. M.P.M and N.N are supported by MRC project grants awarded to M.P.M (G0801242, G1100162).

Received: April 30, 2013
Revised: August 5, 2013
Accepted: September 30, 2013
Published: October 31, 2013

REFERENCES

Beckmann, J., Schubert, R., Chiquet-Ehrismann, R., and Müller, D.J. (2013). Deciphering teneurin domains that facilitate cellular recognition, cell-cell adhesion, and neurite outgrowth using atomic force microscopy-based single-cell force spectroscopy. Nano Lett. *13*, 2937–2946.

Ben Fredj, N., Hammond, S., Otsuna, H., Chien, C.B., Burrone, J., and Meyer, M.P. (2010). Synaptic activity and activity-dependent competition regulates axon arbor maturation, growth arrest, and territory in the retinotectal projection. J. Neurosci. *30*, 10939–10951.

Connaughton, V.P., Behar, T.N., Liu, W.L., and Massey, S.C. (1999). Immunocytochemical localization of excitatory and inhibitory neurotransmitters in the zebrafish retina. Vis. Neurosci. *16*, 483–490.

Dharmaratne, N., Glendining, K.A., Young, T.R., Tran, H., Sawatari, A., and Leamey, C.A. (2012). Ten-m3 is required for the development of topography in the ipsilateral retinocollicular pathway. PLoS ONE *7*, e43083.

Draper, B.W., Morcos, P.A., and Kimmel, C.B. (2001). Inhibition of zebrafish fgf8 pre-mRNA splicing with morpholino oligos: a quantifiable method for gene knockdown. Genesis *30*, 154–156.

Gollisch, T., and Meister, M. (2010). Eye smarter than scientists believed: neural computations in circuits of the retina. Neuron *65*, 150–164.

Hong, W., Mosca, T.J., and Luo, L. (2012). Teneurins instruct synaptic partner matching in an olfactory map. Nature *484*, 201–207.

Kay, J.N., Finger-Baier, K.C., Roeser, T., Staub, W., and Baier, H. (2001). Retinal ganglion cell genesis requires lakritz, a Zebrafish atonal Homolog. Neuron *30*, 725–736.

Kay, J.N., Roeser, T., Mumm, J.S., Godinho, L., Mrejeru, A., Wong, R.O., and Baier, H. (2004). Transient requirement for ganglion cells during assembly of retinal synaptic layers. Development *131*, 1331–1342.

Kenzelmann-Broz, D., Tucker, R.P., Leachman, N.T., and Chiquet-Ehrismann, R. (2010). The expression of teneurin-4 in the avian embryo: potential roles in patterning of the limb and nervous system. Int. J. Dev. Biol. *54*, 1509–1516.

Leamey, C.A., Merlin, S., Lattouf, P., Sawatari, A., Zhou, X., Demel, N., Glendining, K.A., Oohashi, T., Sur, M., and Fässler, R. (2007). Ten_m3 regulates eye-specific patterning in the mammalian visual pathway and is required for binocular vision. PLoS Biol. *5*, e241.

Lowe, A.S., Nikolaou, N., Hunter, P.R., Thompson, I.D., and Meyer, M.P. (2013). A systems-based dissection of retinal inputs to the zebrafish tectum reveals different rules for different functional classes during development. J. Neurosci. *33*, 13946–13956.

Mangrum, W.I., Dowling, J.E., and Cohen, E.D. (2002). A morphological classification of ganglion cells in the zebrafish retina. Vis. Neurosci. *19*, 767–779.

Masland, R.H. (2012). The neuronal organization of the retina. Neuron *76*, 266–280.

Matsuoka, R.L., Nguyen-Ba-Charvet, K.T., Parray, A., Badea, T.C., Chédotal, A., and Kolodkin, A.L. (2011). Transmembrane semaphorin signalling controls laminar stratification in the mammalian retina. Nature *470*, 259–263.

Meyer, M.P., and Smith, S.J. (2006). Evidence from in vivo imaging that synaptogenesis guides the growth and branching of axonal arbors by two distinct mechanisms. J. Neurosci. *26*, 3604–3614.

Mieda, M., Kikuchi, Y., Hirate, Y., Aoki, M., and Okamoto, H. (1999). Compartmentalized expression of zebrafish ten-m3 and ten-m4, homologues of the Drosophila ten(m)/odd Oz gene, in the central nervous system. Mech. Dev. *87*, 223–227.

Mosca, T.J., Hong, W., Dani, V.S., Favaloro, V., and Luo, L. (2012). Trans-synaptic Teneurin signalling in neuromuscular synapse organization and target choice. Nature *484*, 237–241.

Mumm, J.S., Williams, P.R., Godinho, L., Koerber, A., Pittman, A.J., Roeser, T., Chien, C.B., Baier, H., and Wong, R.O. (2006). In vivo imaging reveals dendritic targeting of laminated afferents by zebrafish retinal ganglion cells. Neuron *52*, 609–621.

Nevin, L.M., Robles, E., Baier, H., and Scott, E.K. (2010). Focusing on optic tectum circuitry through the lens of genetics. BMC Biol. *8*, 126.

Nikolaou, N., Lowe, A.S., Walker, A.S., Abbas, F., Hunter, P.R., Thompson, I.D., and Meyer, M.P. (2012). Parametric functional maps of visual inputs to the tectum. Neuron *76*, 317–324.

Osterhout, J.A., Josten, N., Yamada, J., Pan, F., Wu, S.W., Nguyen, P.L., Panagiotakos, G., Inoue, Y.U., Egusa, S.F., Volgyi, B., et al. (2011). Cadherin-6 mediates axon-target matching in a non-image-forming visual circuit. Neuron *71*, 632–639.

Randlett, O., MacDonald, R.B., Yoshimatsu, T., Almeida, A.D., Suzuki, S.C., Wong, R.O., and Harris, W.A. (2013). Cellular requirements for building a retinal neuropil. Cell Rep. *3*, 282–290.

Robles, E., Filosa, A., and Baier, H. (2013). Precise lamination of retinal axons generates multiple parallel input pathways in the tectum. J. Neurosci. *33*, 5027–5039.

Roska, B., and Werblin, F. (2001). Vertical interactions across ten parallel, stacked representations in the mammalian retina. Nature *410*, 583–587.

Sanes, J.R., and Yamagata, M. (2009). Many paths to synaptic specificity. Annu. Rev. Cell Dev. Biol. *25*, 161–195.

Sanes, J.R., and Zipursky, S.L. (2010). Design principles of insect and vertebrate visual systems. Neuron *66*, 15–36.

Tucker, R.P., and Chiquet-Ehrismann, R. (2006). Teneurins: a conserved family of transmembrane proteins involved in intercellular signaling during development. Dev. Biol. *290*, 237–245.

Tucker, R.P., Beckmann, J., Leachman, N.T., Schöler, J., and Chiquet-Ehrismann, R. (2012). Phylogenetic analysis of the teneurins: conserved features and premetazoan ancestry. Mol. Biol. Evol. *29*, 1019–1029.

Wässle, H. (2004). Parallel processing in the mammalian retina. Nat. Rev. Neurosci. *5*, 747–757.

Williams, M.E., de Wit, J., and Ghosh, A. (2010). Molecular mechanisms of synaptic specificity in developing neural circuits. Neuron *68*, 9–18.

Xiao, T., Staub, W., Robles, E., Gosse, N.J., Cole, G.J., and Baier, H. (2011). Assembly of lamina-specific neuronal connections by slit bound to type IV collagen. Cell *146*, 164–176.

Yamagata, M., and Sanes, J.R. (2008). Dscam and Sidekick proteins direct lamina-specific synaptic connections in vertebrate retina. Nature *451*, 465–469.

Yamagata, M., and Sanes, J.R. (2012). Expanding the Ig superfamily code for laminar specificity in retina: expression and role of contactins. J. Neurosci. *32*, 14402–14414.

Young, T.R., Bourke, M., Zhou, X., Oohashi, T., Sawatari, A., Fässler, R., and Leamey, C.A. (2013). Ten-m2 is required for the generation of binocular visual circuits. J. Neurosci. *33*, 12490–12509.

Supplemental Information

Teneurin-3 Specifies Morphological and Functional Connectivity of Retinal Ganglion Cells in the Vertebrate Visual System

Paride Antinucci,[1] Nikolas Nikolaou,[1] Martin P Meyer,[1] and Robert Hindges[1,*]

[1] MRC Centre for Developmental Neurobiology,
King's College London, Guy's Campus,
London SE1 1UL, UK

*Correspondence: robert.hindges@kcl.ac.uk

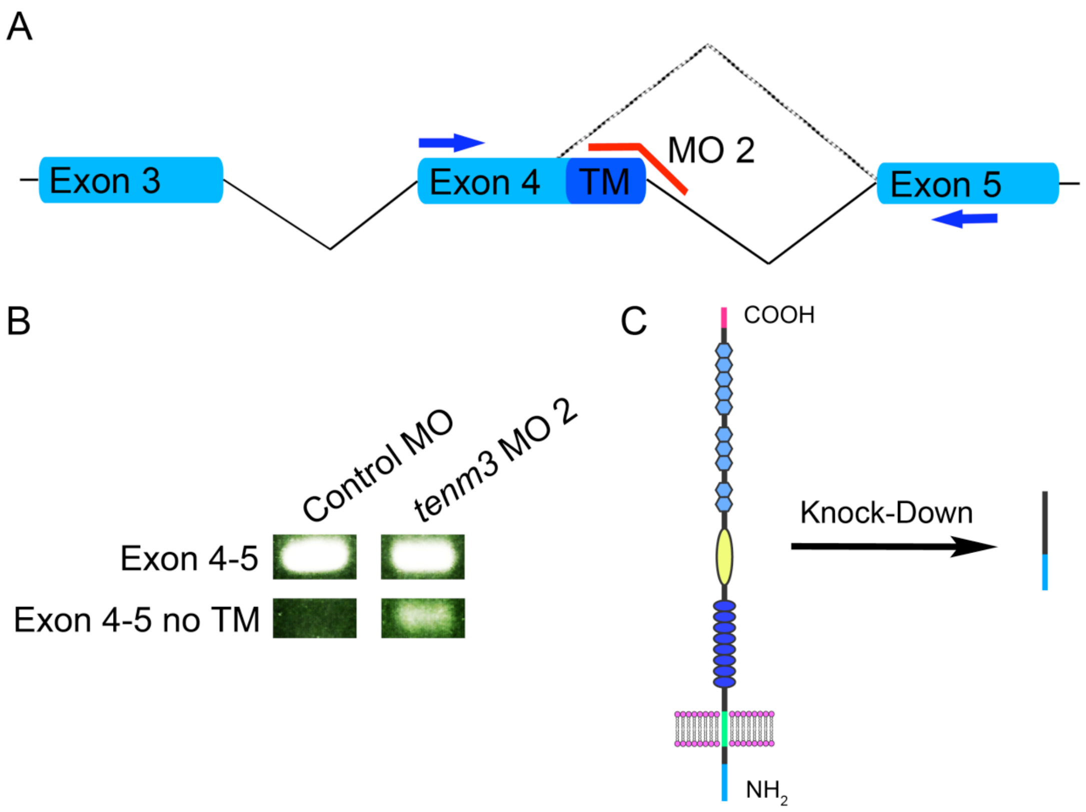

Figure S1. Design and Effect of the Second Splice-Blocking Morpholino Against *teneurin-3*, Related to Figure 1 and S4

(A) Schematic detailing the targeting site of the second splice-blocking *tenm3* morpholino (MO 2), which is shown in red. Importantly, this MO targets a non-overlapping region of *tenm3* mRNA, namely the boundary between exon 4 and intron 4. Exons are represented in cyan. Solid lines indicate introns. The dashed line indicates deletion from exon 4 of the sequence encoding the transmembrane domain (TM, blue) caused by the activation of a cryptic splice donor site following *tenm3* MO 2 injections. Primers used for RT-PCR (B) are reported as blue arrows.

(B) RT-PCR analysis of *tenm3* mRNA structure in control MO- and *tenm3* MO 2-injected embryos. A shorter splice variant is present in *tenm3* morphants. cDNA sequence comparison after DNA sequencing revealed that the short splice variant lacks 64 bp from the 3' region of exon 4. Importantly, this region encodes the transmembrane domain. This deletion leads to a frameshift in exon 5 and a consequent early stop codon in the same exon, resulting in deletion of the transmembrane and extracellular domains.

(C) Schematic detailing the effect of *tenm3* MO 2, which produces the deletion of Tenm3 transmembrane and extracellular domains. The full-length protein is represented on the left. The N-terminus is located intracellularly, whereas the C-terminus is in the extracellular space.

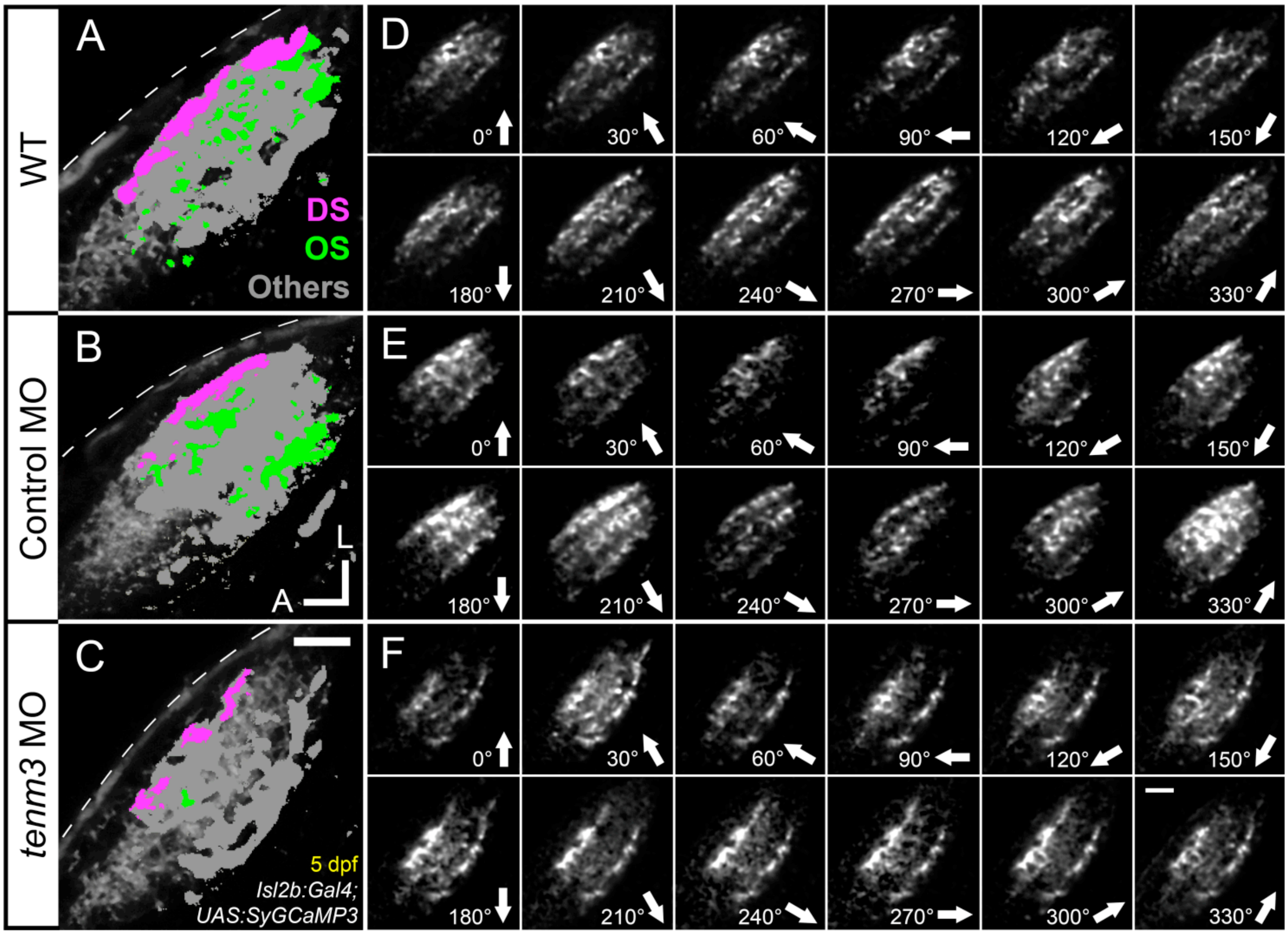

Figure S2. RGC Functional Responses to Drifting Bars, Related to Figure 5 and Movies S1-S3

(A-C) Parametric maps of single 5 dpf Tg(*Isl2b:Gal4;UAS:SyGCaMP3*) larvae (one larva per animal group) representing the spatial distribution of direction-selective (DS, magenta), orientation-selective (OS, green) and other visually responsive voxels (others, grey) superimposed onto the mean fluorescence images of SyGCaMP3-expressing axons (greyscale). Note that the *tenm3* morphant larva (C) shows substantially less OS voxels than WT and control MO-injected larvae (A and B). Dashed lines indicate the skin overlaying the tectum.

(D-E) Montages showing integral responses (grayscale) of all voxels (RGC axons expressing SyGCaMP3) in the tectal neuropil of the larvae described in (A-C). Note that all three larvae exhibit complex response patterns to moving bars. Direction of motion is shown on the bottom right in each panel. Scale bars = 20 µm. A, anterior; L, lateral. The three representative larvae shown in (A-E) are the same used to generate Movies S1-S3.

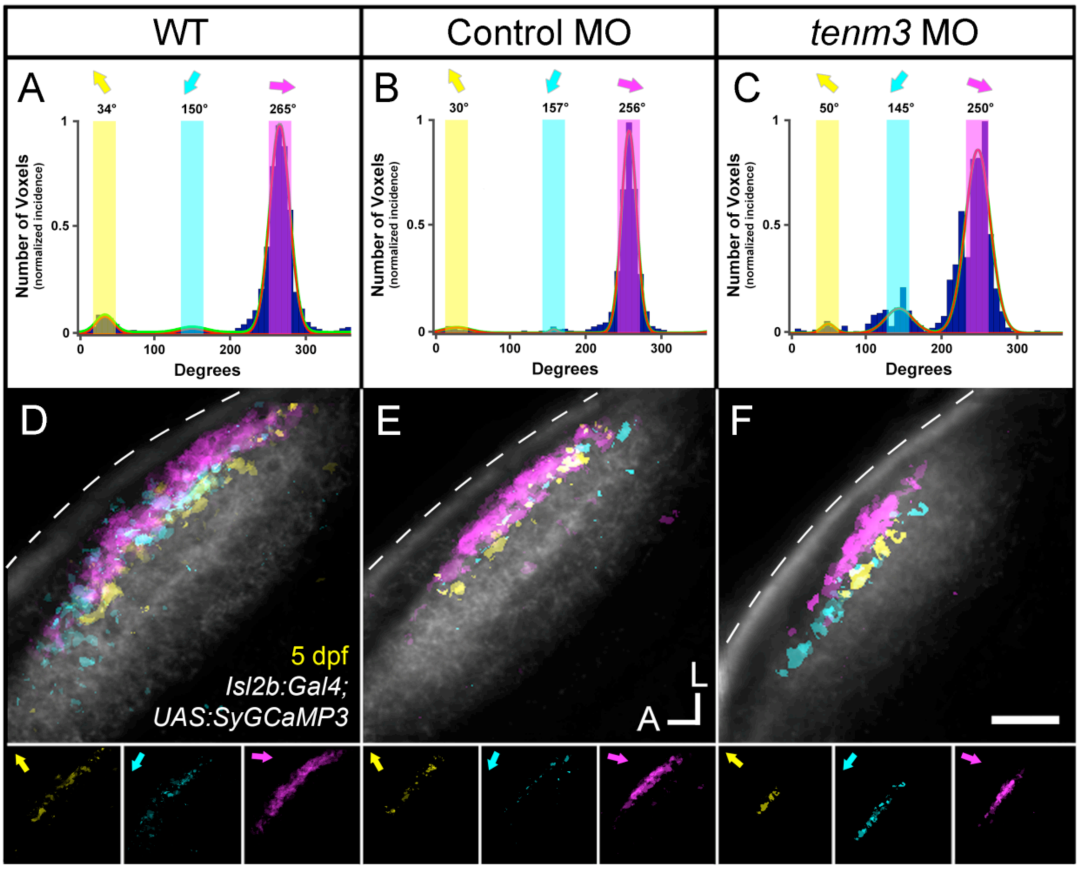

Figure S3. The Three Direction-Selective RGC Subtypes Are Not Affected by *teneurin-3* Knock-Down, Related to Figure 5

(A-C) Cumulative histograms summarizing the incidence of direction-selective (DS) voxels within each group (WT n = 8 larvae; control MO n = 11; *tenm3* MO n = 20). Fitted von-Mises distributions reveal three populations of DS voxels tuned to three different directions of motion. Individual peak preferred angles are reported above. Note that all three animal groups develop three DS RGC subtypes. Moreover, the sizes of individual DS voxel populations are comparable across groups, with the dominant input corresponding to anterior motion selectivity.

(D-F) Composite parametric maps across multiple 5 dpf larvae representing the spatial distribution of the three DS voxel subtypes within each group (WT n = 8 larvae; control MO n = 11; *tenm3* MO n = 20). Note that the laminar organization of the three DS voxel subtypes in the tectal neuropil is analogous in all three groups, namely they are confined to superficial layers of the SFGS. Individual parametric maps for each voxel subtype are shown at the bottom. Color coding as per (A-C). The standard space template image derived for each group (greyscale) provides an anatomical reference. Dashed lines indicate the skin overlaying the tectum. Scale bar = 20 μm. A, anterior; L, lateral.

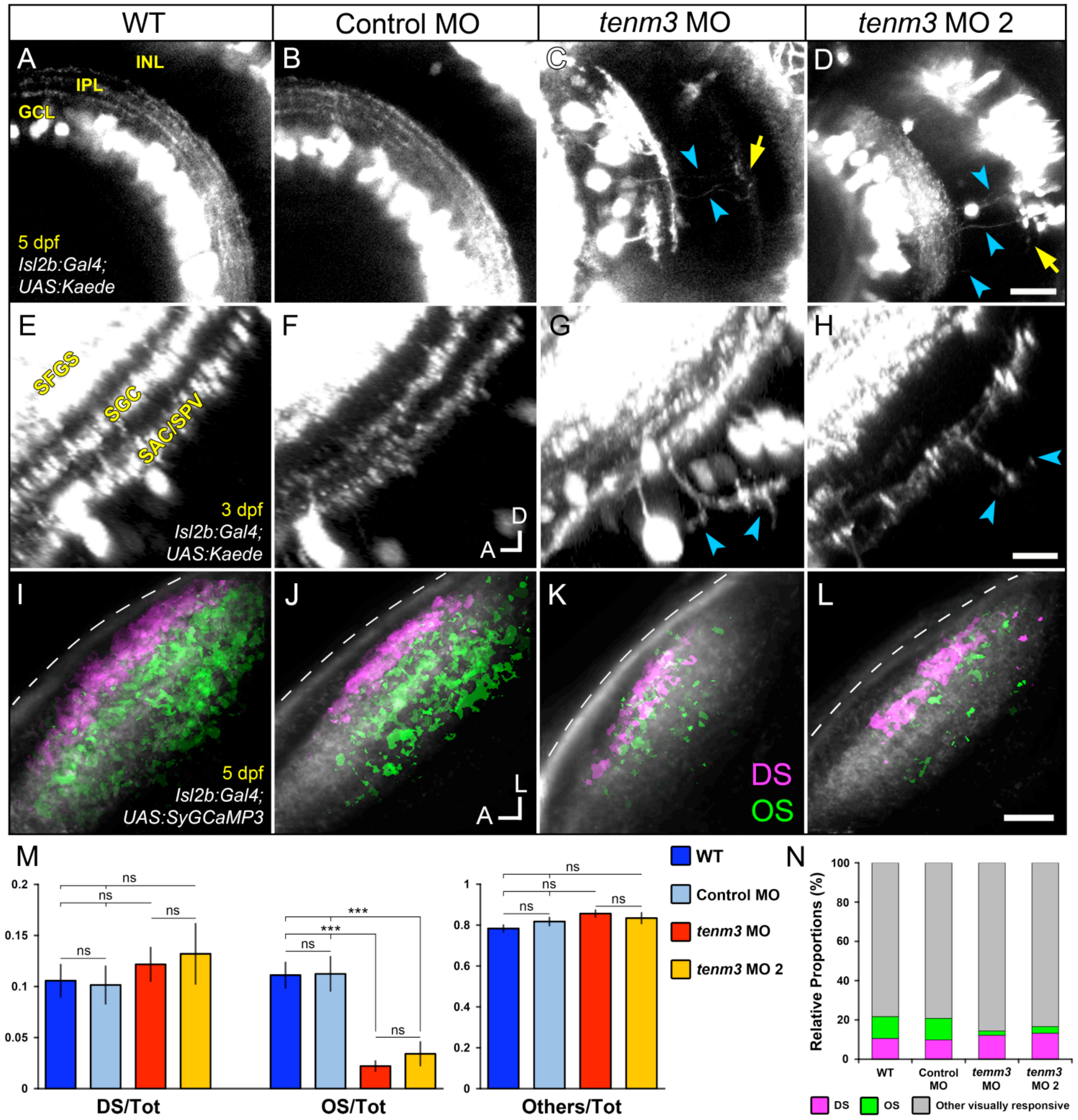

Figure S4. Two Different Morpholino Oligonucleotides Against *teneurin-3* Produce Equivalent Structural and Functional Results, Related to Figure 2, 4, 5 and S1

(A-D) Kaede-expressing RGCs in the retina of 5 dpf WT, control MO-, *tenm3* MO-, and *tenm3* MO 2-injected Tg(*Isl2b:Gal4;UAS:Kaede*) zebrafish larvae. Aberrant RGC neurite extensions in the inner nuclear layer (INL) are detected in *tenm3* MO-injected larvae (C) as well as in *tenm3* MO 2-injected larvae (D, cyan arrowheads) but not in WT (A) and Control MO-injected (B) larvae. In some cases these processes reach the outer plexiform layer both in *tenm3* MO- (C) and *tenm3* MO 2-injected larvae (D, yellow arrows) but not in control larvae. All images represent maximum intensity projections of ~20 µm confocal Z stacks. Scale bar = 20 µm. GCL, ganglion cell layer; IPL, inner plexiform layer. WT, control MO and *tenm3* MO images in Figure 2 are reported here for comparison.

(E-H) RGC axon lamination in deep laminae of the tectal neuropil of 3 dpf Tg(*Isl2b:Gal4;UAS:Kaede*) zebrafish larvae. Aberrant extensions projecting into the stratum periventriculare (SPV) are detected in *tenm3* MO-injected larvae (G) as well as in *tenm3* MO 2-injected larvae (H, cyan arrowheads) but not in control larvae (E and F). All images represent maximum intensity projections of ~50 µm confocal Z stacks that have been rotated around the longitudinal axis to best show axonal lamination. Scale bar = 10 µm. A, anterior; D, dorsal. SFGS, stratum fibrosum et griseum superficiale; SAC, stratum album centrale. WT, control MO and *tenm3* MO images in Figure 4 are reported here for comparison.
(I-L) Composite parametric maps across multiple 5 dpf Tg(*Isl2b:Gal4;UAS:SyGCaMP3*) larvae representing the spatial distribution of direction-selective (DS, magenta) and orientation-selective (OS, green) voxels within each group (WT n = 8 larvae; control MO n = 11; *tenm3* MO n = 20; *tenm3* MO 2 n = 8). Note that both in *tenm3* MO- (K) and *tenm3* MO 2-injected larvae (L) the overall number of OS voxels is decreased compared to control larvae (I and J). Within individual parametric maps, voxel brightness is proportional to the summed incidence of each functional response across all larvae imaged. The standard space template image derived for each group (greyscale) provides an anatomical reference. Dashed lines indicate the skin overlaying the tectum. Scale bar = 20 µm. A, anterior; L, lateral. WT, control MO and *tenm3* MO images in Figure 5 are reported here for comparison.
(M) Ratios between defined voxel classes and total visually responsive voxels within each group. Both in *tenm3* MO- and *tenm3* MO 2-injected larvae there is a significant change in the ratio between OS voxels and the total population of visually responsive voxels (OS/tot *tenm3* MO 0.022 ± 0.004, n = 20 larvae; *tenm3* MO 2 0.034 ± 0.011, n = 8; WT 0.111 ± 0.012, n = 8; control MO 0.112 ± 0.016, n = 11; $F_{(3,44)} = 18.88$, $p < 0.0001$). No significant change was observed in the relative proportions of the other voxel populations (DS/tot WT 0.105 ± 0.015, control MO 0.101 ± 0.018, *tenm3* MO 0.121 ± 0.016, *tenm3* MO 2 0.132 ± 0.029, $F_{(3,44)} = 0.37$, $p = 0.775$; Others/tot WT 0.783 ± 0.016, control MO 0.817 ± 0.019, *tenm3* MO 0.856 ± 0.016, *tenm3* MO 2 0.833 ± 0.025, $F_{(3,44)} = 1.999$, $p = 0.128$, n = 47 larvae). Non-DS and non-OS voxels are classified as 'others'. All graphs show mean values ± SEM. ***p < 0.001; ns, not significant by one-way ANOVA followed by Tukey's HSD test.
(N) Bar graph showing the proportions of DS and OS voxel classes relative to visually responsive voxels within each group. Note that the OS input becomes the smallest population of RGCs responding to drifting bars both in *tenm3* MO- and *tenm3* MO 2-injected larvae.

SUPPLEMENTAL MOVIE TITLES AND LEGENDS

Movie S1. Responses of SyGCaMP3-Expressing Axons in the Tectal Neuropil of a WT Larva Evoked by Drifting Bars, Related to Figure 5 and S2

Time-lapse functional data of a representative 5 dpf WT larva. The movie encompasses an entire tuning experiment in which all 12 directions of bar motion plus a blank screen null stimulus are presented to one eye of the immobilized zebrafish larva. Unprocessed SyGCaMP3 responses are shown on the left, whereas ΔF/F responses are reported on the right. Time given in min:sec. The same data are presented as parametric map and montage in Figures S2A and S2D, respectively.

Movie S2. Responses of SyGCaMP3-Expressing Axons in the Tectal Neuropil of a Control MO-Injected Larva Evoked by Drifting Bars, Related to Figure 5 and S2

Time-lapse functional data of a representative 5 dpf control MO-injected larva. The movie encompasses an entire tuning experiment in which all 12 directions of bar motion plus a blank screen null stimulus are presented to one eye of the immobilized zebrafish larva. Unprocessed SyGCaMP3 responses are shown on the left, whereas ΔF/F responses are reported on the right. Time given in min:sec. The same data are presented as parametric map and montage in Figures S2B and S2E, respectively.

Movie S3. Responses of SyGCaMP3-Expressing Axons in the Tectal Neuropil of a *teneurin-3* Morphant Larva Evoked by Drifting Bars, Related to Figure 5 and S2

Time-lapse functional data of a representative 5 dpf *tenm3* morphant larva. The movie encompasses an entire tuning experiment in which all 12 directions of bar motion plus a blank screen null stimulus are presented to one eye of the immobilized zebrafish larva. Unprocessed SyGCaMP3 responses are shown on the left, whereas ΔF/F responses are reported on the right. Time given in min:sec. The same data are presented as parametric map and montage in Figures S2C and S2F, respectively.

EXTENDED EXPERIMENTAL PROCEDURES

Animals

Zebrafish were maintained at 28.5°C on a 14 hr ON/10 hr OFF light cycle in Danieau solution [58 mM NaCl, 0.7 mM KCl, 0.4 mM $MgSO_4$, 0.6 mM $Ca(NO_3)_2$ 5.0 mM HEPES, pH 7.6]. The AB strain of zebrafish was used for *in situ* hybridization, RT-PCR, visual background adaptation and mosaic labeling of retinal ganglion cells (RGCs). Transgenic lines used in this study include Tg(*Isl2b:Gal4*) (Ben Fredj et al., 2010), Tg(*UAS:Kaede*) (gift of Prof. Chi Bin-Chien) and Tg(*UAS:SyGCaMP3*) (Nikolaou et al., 2012). Functional imaging experiments were performed in the pigmentation mutant *nacre,* which lacks all neural crest derived melanophores (Lister et al., 1999). Larvae used for all the other experiments were raised in 0.003% phenylthiourea (Sigma) in 1x Danieau solution to avoid pigment formation. This work was approved by the local Animal Care and Use Committee (King's College London), and was carried out in accordance with the Animals (Experimental Procedures) Act, 1986, under licence from the United Kingdom Home Office.

In Situ Hybridization

To make the *tenm3* antisense riboprobe, a 981 bp cDNA fragment (ORF 7034-8014) was cloned into a StrataClone Blunt PCR Cloning Vector pSC-B-amp/kan (Agilent Technologies), and the orientation of the insertion was determined by DNA sequencing. The sequences of primers used to amplify the fragment through PCR are as follows: forward primer 5'-GGGACTATGACATTCAAG CAGGTC-3'; reverse primer 5'-CATTGTTGGCACTGTCGGCCAG-3'. The antisense RNA probe was generated from the linearized plasmid using T3 RNA polymerase (Life Technologies) and digoxigenin-labeled nucleotides (Roche). Whereas, the sense digoxigenin-labeled RNA probe was generated using T7 RNA polymerase (Life Technologies). After synthesis, riboprobes were treated with DNase I (Ambion) for 15 minutes at 37°C.

The same protocol described in Thisse and Thisse (2008) was used to perform whole-mount *in situ* hybridisations. Briefly, embryos were fixed in 4% paraformaldehyde (PFA) in PBS overnight at 4°C, dehydrated in methanol and stored at -20°C. Subsequently, embryos were rehydrated in 75% methanol in PBT (0.1% Tween 20 in PBS), 50% methanol in PBT, 25% methanol in PBT, and PBT for 5 minutes each. 2, 3 and 5 dpf embryos were digested with proteinase K (10 µg/ml; Sigma) at room temperature for 20, 30 and 60 minutes, respectively. Subsequently, they were fixed in 4% PFA in PBS for 20 minutes at room temperature and washed several times in PBT. They were then transferred to hybridisation mix (HM; 50% formamide, 5x SSC, 0.1% Tween 20, 50 µg/ml heparin, 0.5 mg/ml yeast tRNA, and 9 mM citric acid to pH 6.0 in DEPC ddH_2O) and incubated for 4 hours at 65°C. The HM was replaced with a solution containing 1 µg/ml of digoxigenin-labeled RNA probe in HM and the embryos were incubated overnight at 65°C. Washes were performed at the hybridisation temperature in 65% HM/35% 2x SSC, 35% HM/65% 2x SSC, 2x SSC, for 10 minutes each and, finally, in 0.2x SSC + 0.1% Tween 20 for 20 minutes and 2 times 20 minutes each in 0.1x SSC + 0.1% Tween 20. A series of washes were performed at room temperature in 65% 0.2x SSC/35% PBT, 35% 0.2x SSC/65% PBT, and PBT for 10 minutes each. Embryos were then incubated in blocking solution (5% sheep serum in PBT) for 4 hours at room temperature. The blocking solution was replaced with alkaline phosphatase-conjugated anti-digoxigenin Fab fragments (Roche) diluted 1:2000 in blocking solution and embryos were incubated at 4°C overnight. After washing at least 8 times 1 hour each in PBT at room temperature followed by

overnight wash in PBT at 4°C, embryos were rinsed 3 times 20 minutes each in NTMT staining buffer (0.1 M Tris-HCl pH 9.5, 50 mM $MgCl_2$, 0.1 M NaCl, 0.1% Tween 20). The staining buffer was then replaced with 2% NBT/BCIP stock solution (Roche) in NTMT and embryos were incubated in the dark at room temperature. To stop the reaction, embryos were washed in PBS and fixed in 4% PFA in PBS for 20 minutes at room temperature. After several washes in PBS, embryos were washed in 15% and 30% sucrose in PBS for 2 hours each at room temperature, then in 40% sucrose in PBS overnight at 4°C. Subsequently, embryos were embedded in OCT (VWR International) and frozen in dry ice. Finally, 20 µm-thick sections were cut using a cryostat and stored in 70% glycerol in PBS.

Morpholino and DNA Microinjections

2-2.5 ng/1.8 nl (0.24-0.3 pmols) of morpholino oligonucleotides (MOs; Gene Tools) in Danieau solution were injected into one-cell stage zebrafish embryos. The sequences of MOs used are as follows: splice-blocking *tenm3* MO 5'-ACGGTTGCTctgtggaaaaaaatca-3' (intronic sequence in lower case); splice-blocking *tenm3* MO 2 5'-taggcagtgttaaacttacCAATGC-3'; standard control MO 5'-CCTCTTACCTCAGTTACAATTTATA-3'.

In order to mosaically label RGCs, an activator plasmid containing Gal4 driven by an upstream *ath5* promoter (*Ath5:Gal4*) (gift of Prof. Steve Wilson, UCL, UK) was co-injected with effector plasmids, where expression is driven by a UAS motif in frame with either GFP (*UAS:GFP*) or tdTomato (*UAS:tdTomato*) (Ben Fredj et al., 2010). The plasmids were injected at a concentration of 20 ng/µl each in Danieau solution. Plasmid DNA was prepared using miniprep kits (Qiagen).

RT-PCR

Total RNA was isolated from multiple dechorionated embryos (~30) using TRIzol reagent (Life Technologies). cDNA was synthesized using SuperScript III reverse transcriptase (Life Technologies) and random primers (Promega, C1181). The obtained cDNA was then used as template for amplification through PCR. For *tenm3* MO, the sequences of the primers are as follows: forward primer 5’-ATGCCATCCTCTCTCTCCAGTCCA-3’; reverse primer 5’-ACTTC TTGAACTTGAAGGCGCTGC-3’. They target exon 2 and exon 4 of the tenm3-001 splice variant, respectively. Whereas, for *tenm3* MO2 the sequences of the primers are as follows: forward primer 5’-CGGCCACGCCAGGCTACACTATG-3’; reverse primer 5’-TCCTGCAGCTGCCAATTCAGTCC-3’. They target exon 4 and exon 5 of the tenm3-001 splice variant, respectively. Full-length and shorter splice variants were extracted from agarose gel using QIAquick Gel Extraction Kits (Qiagen), cloned into StrataClone Blunt PCR Cloning Vectors pSC-B-amp/kan (Agilent Technologies) and verified by sequencing.

Visual Background Adaptation

Visual background adaptation of 4 dpf larvae was assayed by exposure to bright light for more than 3 hours, followed by visual inspection of pigmentation using a stereomicroscope.

Imaging

Imaging was performed using an LSM 710 confocal microscope equipped with a spectral detection scan head and a 20x/1.0 NA water-immersion objective (Carl Zeiss). Functional time-series of visually-evoked SyGCaMP3 responses were acquired at a rate of 4.1 Hz and 0.415x0.415 µm resolution (256x256 pixels) and 1 AU pinhole aperture. The average diameter of a presynaptic bouton in zebrafish RGCs is ~0.8 µm (Meyer and Smith, 2006). Thus, the physical X-Y dimensions of voxels are below that of a typical presynaptic bouton. Excitation was provided by 488 nm multi-

line laser. Optical sections were obtained at <1.6 μm intervals, and maximum intensity projections of Kaede-, GFP- and tdTomato-positive neurons were generated using either ImageJ (NIH) or ZEN (Carl Zeiss). Imaging of cryosections after *in situ* hybridizations was performed using a Zeiss Axioskop microscope connected to a cooled monochrome CCD camera (Retiga EXi Blue) and Volocity acquisition software (PerkinElmer).

Data Analysis

All images were processed using either ImageJ (NIH) or ZEN (Carl Zeiss). To make Figure 2E, *a* rectangular region of interest was drawn across a short, relatively straight stretch of the IPL with pronounced dendrite stratification. The Plot Profile function in ImageJ (NIH) was applied to the rectangle to calculate the fluorescence intensity trace across the IPL width. The values obtained from multiple larvae were then normalized and averaged using SigmaPlot (Systat Software). Analyses of IPL width and RGC axon thickness, length and branching were carried out using ImageJ (NIH).

Visual Stimulation and Voxel-Wise Analysis

Visual stimulation was performed as previously described (Nikolaou et al., 2012). In summary, larvae with SyGCaMP3 expression in RGC presynaptic terminals, were restrained in 2% low melting point agarose (Sigma) in 1x Danieau solution, mounted with the dorsal side up onto a customized glass platform and placed in a Danieau-filled chamber. The agarose was sufficient to restrain the larvae so that anesthesia was not required. The agarose surrounding one eye was removed allowing an unobstructed view of the projected image on one side of the chamber, which served as a projection screen. Time-series were recorded from the contralateral tectal neuropil. The projected image filled a visual field of approximately 97° by 63°. Visual stimuli consisted of light (56 cd/m^2) or dark bars (8 cd/m^2) (175% and 25% of mean, respectively) on a mean gray background (32 cd/m^2). Since no qualitative differences between light and dark bars responses were noted, data for the two stimuli were combined. Each bar was 10° in width moving at 20°/s and separated from the preceding bar by 30°, therefore enabling more than one bar on the screen at any one time. The long axis of the bar was orthogonal to the direction of motion. Bars were presented at 12 different directions evenly spaced across 360° and displayed in a pseudo-random order. A blank screen null condition of 2 s was also interleaved. Each inter-epoch interval was 8 s to enable the SyGCaMP3 signal to return baseline. Visual experiments were generated and controlled using custom written Labview and Matlab code (MathWorks) implemented on a ViSaGe stimulus presenter (Cambridge Research Systems, UK) and delivered via a DLP picoprojector (Optoma).

Voxel-wise analysis of direction- and orientation-selective responses and generation of composite maps were performed as previously described (Nikolaou et al., 2012) excepting that a goodness of fit (R^2) was used in the identification of functional classes of voxels (Lowe et al., 2013). Briefly, a threshold for each voxel within an acquisition image was determined from the variance of ΔF changes during the inter-epoch intervals and null condition – threshold ≡ 5 standard deviations. All voxels that were supra-threshold within at least two visually presentation epochs were regarded as visually responsive and subjected to further characterization: direction selectivity and orientation selectivity. Direction- and orientation-selective indices (DSI and OSI) (Niell and Stryker, 2008) based on fitted von-Mises profiles (Swindale, 1998) were calculated together with an estimate for their goodness of fit – R^2 (Lowe et al., 2013). To minimize cross-talk and over-fitting associated with DSI and OSI metrics a stringent approach was undertaken. For a voxel to be regarded as DS or OS mutually exclusive criteria were employed: DS if DSI > 0.5 and OSI < 0.5; and OS if OSI > 0.5 and

DSI < 0.5. In both cases the goodness of fit for DSI and OSI respectively had to be greater than 0.8, thus the fitted curves explained at least 80% of the integral responses.

Statistical Analyses

The statistical significance of the differences between mean values among animal groups was determined by one-way analysis of variance (ANOVA) followed by Tukey's HSD test, using SigmaPlot (Systat Software). The criterion for statistical significance was set at $p < 0.05$ and all results are represented as mean ± SEM. The statistical significance of the difference in the proportion of diffuse RGCs among groups was determined by chi-squared test, using SigmaPlot (Systat Software). The criterion for statistical significance was set at $p < 0.05$.

SUPPLEMENTAL REFERENCES

Ben Fredj, N., Hammond, S., Otsuna, H., Chien, C.B., Burrone, J., and Meyer, M.P. (2010). Synaptic activity and activity-dependent competition regulates axon arbor maturation, growth arrest, and territory in the retinotectal projection. J. Neurosci. *30*, 10939-10951.

Lister, J.A., Robertson, C.P., Lepage, T., Johnson, S.L., and Raible, D.W. (1999). nacre encodes a zebrafish microphthalmia-related protein that regulates neural-crest-derived pigment cell fate. Development *126*, 3757-3767.

Lowe, A.S., Nikolaou, N., Hunter, P.R., Thompson, I.D., and Meyer, M.P. (2013). A systems-based dissection of retinal inputs to the zebrafish tectum reveals different rules for different functional classes during development. J. Neurosci. *33*, 13946-13956.

Meyer, M.P., and Smith, S.J. (2006). Evidence from in vivo imaging that synaptogenesis guides the growth and branching of axonal arbors by two distinct mechanisms. J. Neurosci. *26*, 3604-3614.

Niell, C.M., and Stryker, M.P. (2008). Highly selective receptive fields in mouse visual cortex. J. Neurosci. *28*, 7520-7536.

Nikolaou, N., Lowe, A.S., Walker, A.S., Abbas, F., Hunter, P.R., Thompson, I.D., and Meyer, M.P. (2012). Parametric functional maps of visual inputs to the tectum. Neuron *76*, 317-324.

Swindale, N.V. (1998). Orientation tuning curves: empirical description and estimation of parameters. Biol. Cybern. *78*, 45-56.

Thisse, C., and Thisse, B. (2008). High-resolution in situ hybridization to whole-mount zebrafish embryos. Nat. Protoc. *3*, 59-69.
